# Incidence of invasive pneumococcal disease in children with commercial insurance or Medicaid coverage in the United States before and after the introduction of 7- and 13-valent pneumococcal conjugate vaccines during 1998–2018

**DOI:** 10.1186/s12889-022-14051-6

**Published:** 2022-09-05

**Authors:** Tianyan Hu, Yan Song, Nicolae Done, Qing Liu, Eric M. Sarpong, Esteban Lemus-Wirtz, James Signorovitch, Salini Mohanty, Thomas Weiss

**Affiliations:** 1 Merck & Co., Inc., 126 East Lincoln Ave, P.O. Box 2000, Rahway, NJ 07065 USA; 2 Analysis Group, Inc., 111 Huntington Avenue 14th Floor, Boston, MA 02199 USA

**Keywords:** Incidence rate estimates, Invasive pneumococcal disease, Pneumococcal conjugate vaccines

## Abstract

**Background:**

Invasive pneumococcal disease (IPD) is a major cause of pediatric morbidity and mortality. Pneumococcal conjugate vaccines (PCVs) were introduced in the US in 2000 (PCV7) and 2010 (PCV13). This study estimated the annual incidence rates (IRs) and time trends of IPD to quantify the burden of disease in children before and after the introduction of PCV7 and PCV13 in the US.

**Methods:**

IPD episodes were identified in the IBM MarketScan Commercial and Medicaid Databases using claims with International Classification of Diseases 9/10^th^ Revision, Clinical Modification codes. Annual IRs were calculated as the number of IPD episodes/100,000 person-years (PYs) for children < 18 years and by age group (< 2, 2–4, and 5–17 years). National estimates of annual IPD IRs were extrapolated using Census Bureau data. Interrupted time series (ITS) analyses were conducted to assess immediate and gradual changes in IPD IRs before and after introduction of PCV7 and PCV13.

**Results:**

In commercially insured children, IPD IRs decreased from 9.4 to 2.8 episodes/100,000 PY between the pre-PCV7 (1998–1999) and late PCV13 period (2014–2018) overall, and from 65.6 to 11.6 episodes/100,000 PY in children < 2 years. In the Medicaid population, IPD IRs decreased from 11.3 to 4.2 episodes/100,000 PY between the early PCV7 (2001–2005) and late PCV13 period overall, and from 42.6 to 12.8 episodes/100,000 PY in children < 2 years. The trends of IRs for meningitis, bacteremia, and bacteremic pneumonia followed the patterns of overall IPD episodes. The ITS analyses indicated significant decreases in the early PCV7 period, increases in the late PCV7 and decreases in the early PCV13 period in commercially insured children overall. However, increases were also observed in the late PCV13 period in children < 2 years. The percentage of cases with underlying risk factors increased in both populations.

**Conclusions:**

IRs of IPD decreased from 1998 to 2018, following introduction of PCV7 and PCV13, with larger declines during the early PCV7 and early PCV13 periods, and among younger children. However, the residual burden of IPD remains substantial. The impact of future PCVs on IPD IRs will depend on the proportion of vaccine-type serotypes and vaccine effectiveness in children with underlying conditions.

**Supplementary Information:**

The online version contains supplementary material available at 10.1186/s12889-022-14051-6.

## Background

*Streptococcus pneumoniae* is a leading cause of morbidity, mortality, and healthcare resource utilization worldwide [1, 2]. In the United States (US), *S. pneumoniae* caused about 4 million disease episodes resulting in $3.5 billion in direct medical costs in 2004 [3, 4]. Invasive pneumococcal disease (IPD), including meningitis, bacteremia, and bacteremic pneumonia, is defined as isolation of *S. pneumoniae* from a normally sterile site (e.g., blood or cerebrospinal fluid). In children aged < 2 years, the most common presentation of IPD is bacteremia without focus [5, 6]. In children of all ages, *S. pneumoniae* is a leading cause of bacterial pneumonia and bacterial meningitis [7]. At least 100 serotypes of *S. pneumoniae* have been identified, which are determined by the surface capsular polysaccharide [8–10].

The first pneumococcal conjugate vaccine (PCV) was licensed in the US and recommended by the Advisory Committee on Immunization Practices (ACIP) in 2000, as part of the pediatric vaccine schedule, leading to a rapid increase in vaccination coverage rate among infants [11]. It was a 7-valent PCV (PCV7) and included 7 of the most common serotypes to cause IPD. Over time, IPD caused by vaccine serotypes declined, and increases in non-vaccine serotypes were reported, representing serotype replacement of non-vaccine for vaccine serotypes [12–14]. In 2010, the 13-valent PCV (PCV13), which protects against 6 additional serotypes, replaced PCV7 in the pediatric vaccine schedule. Data from the Centers for Disease Control and Prevention demonstrates that vaccination coverage for the primary series (≥ 3 doses) reached at least 50% by 2003 and exceeded 90% after 2007; coverage for the full series (≥ 4 doses) reached at least 50% by 2005, and exceeded 80% after 2007 [15].

Multiple systematic reviews have demonstrated that PCV is effective in preventing vaccine-type IPD among children [16–18]. Moreover, several studies based on real-world data, specifically national surveillance data, have found significant reductions in the incidence of IPD among children after the introduction of PCV7 and PCV13 [3, 19–21]. Though most studies documented the reduction of IPD incidence during a relatively short period (less than 10 years), Lapidot et al. computed IPD cases and related deaths averted over the whole timeline from the pre-PCV7 period to the post-PCV period, using Active Bacterial Core surveillance (ABCs) data [22]. However, they did not report IRs of IPD over time.Wasserman et al. estimated the number of IPD cases from 1997 to 2017 among US children based on a modelling approach, using population data from the US Census Bureau, IPD incidence from the ABCs data, and existing literature [23]. Both of these studies found substantial declines in annual cases (overall and by IPD syndrome) in children < 5 years of age, but did not conduct analyses of IR trends over time. Furthermore, the incidence trends of IPD and specific IPD syndromes, have not been comprehensively examined over the 20-year timeline using claims data, rather than surveillance reports, and thus a thorough analysis of the burden of pediatric IPD on the healthcare system, including a comparison between the commercially insured and Medicaid populations, is not available [24].

Despite evidence of significant declines in incidence rates (IRs) of IPD after the introduction of PCVs, residual disease caused by persistent vaccine-type serotypes and non-vaccine serotypes remains [6, 25, 26]. Furthermore, two new vaccines were recently approved by the Food and Drug Administration for the prevention of invasive and non-invasive pneumococcal disease in adults, and their efficacy and safety profiles among children are under evaluation in clinical trials [27–31]. It is therefore important to quantify the burden of pediatric IPD comprehensively before and after the introduction of these newly-approved, higher valent PCVs.

The objectives of this study were to evaluate the annual incidence of IPD and its manifestations to quantify the burden of IPD in children under 18 years of age in the US before and after the introduction of PCV7 and PCV13 (1998 to 2018), and to assess changes in the temporal trends of IRs for IPD before and after the introduction of PCV7 and PCV13.

## Methods

This study was conducted using data from the IBM MarketScan® Commercial Claims and Encounters (CCAE; January 1, 1998 to December 31, 2018) and Multi-State Medicaid databases (January 1, 2001 to December 31, 2018). The CCAE database contains enrollment eligibility, demographic data, drug utilization and expenditure data for approximately 90 million employees, as well as their spouses and dependents who are covered by employer-sponsored private health insurance. The Multi-State Medicaid Database contains similar data for nearly 16 million Medicaid enrollees from 12 states. Both databases include information on inpatient, outpatient, and long-term care services. The total population of children (age < 18 years) included in these databases were used to calculate the total number of person-years (PY) at risk, whether or not they had a medical claim.

### Study design and patient population

This retrospective cohort study includes administrative claims data for children under 18 years of age who were enrolled in commercial or Medicaid plans in the United States at any time from January 1998 to December 2018. Children with an IPD episode were identified and defined in each calendar year using claims with International Classification of Diseases 9/10^th^ Revision, Clinical Modification (ICD-9-CM and ICD-10-CM) codes. IPD was defined as pneumococcal-specific meningitis, bacteremia, bacteremic pneumonia, or other pneumococcal-specific IPDs (e.g., arthritis, peritonitis, pericarditis, endocarditis, and osteomyelitis).

The 21-year study period was sub-divided into 5 periods, based on the timing of introduction and the number of serotypes in the vaccine: (1) pre-PCV7 (1998–1999); (2) early PCV7 (2001–2005); (3) late PCV7 (2006–2009); (4) early PCV13 (2011–2013); and (5) late PCV13 (2014–2018). The years 2000 and 2010 were considered transitional years, as these were the years when the PCV7 and PCV13 vaccines were introduced, and thus were excluded from the time trend analysis.

### Study outcomes

#### Invasive pneumococcal disease episodes

IPD episodes were identified through inpatient and outpatient claims by the presence of ICD-9 or ICD-10 diagnosis codes, which were identified in the primary positions of inpatient claims and in the primary or secondary positions of outpatient claims. In the main analysis, four IPD manifestations were separately identified. Pneumococcal-specific meningitis was identified using an ICD-9/10 code for pneumococcal meningitis, or a combination of a code for unspecified meningitis and a code for pneumococcal infection. Pneumococcal-specific bacteremia was identified using an ICD-9/10 code for pneumococcal bacteremia, or a combination of a code for unspecified bacteremia and a code for pneumococcal infection. Pneumococcal-specific bacteremic pneumonia was identified using one of the following: a combination of a code for empyema or abscess of lung and a code for pneumococcal infection; or a combination of a code for pneumococcal bacteremia and a code for all-cause pneumonia (pneumonia due to any bacterial or viral cause); or a combination of a code for unspecified bacteremia, a code for pneumococcal infection, and a code for all-cause pneumonia; or a combination of a code for unspecified bacteremia and a code for pneumococcal-specific pneumonia. Other pneumococcal-specific IPDs, including conditions not listed above (e.g., arthritis, peritonitis, pericarditis, endocarditis, and osteomyelitis), were identified using an ICD-9/10 code for a pneumococcal-specific condition, or a combination of a code for an unspecified condition and a code for pneumococcal infection. See Supplemental Table A1 for the specific codes used for meningitis, bacteremia, bacteremic pneumonia, and other IPDs.

An IPD episode was defined as one or more outpatient and/or inpatient claims, with a gap of at least 90 days with no IPD-related diagnoses required to define the start of a new episode. When diagnosis codes present on the same claim fulfilled the definitions for multiple manifestations, the higher-priority manifestation took precedence according to the following hierarchy to avoid double-counting: meningitis > bacteremia > bacteremic pneumonia > other IPD. The index date was defined as the service date of the first claim during each episode, and was used to assign episodes to a calendar year. Episodes spanning two different years were assigned to the year in which the index claim occurred.

#### Risk factors

Risk factors that predispose children to pneumococcal infection were also identified in the 6-month time window prior to the start of the index episode, which was defined as the first episode for each patient in each calendar year. The risk factor definitions were based on the US Centers for Disease Control and Prevention and the ACIP recommendation for pneumococcal vaccination in high-risk children [32, 33].

### Statistical analysis

#### Analysis of incidence rates

Annual incidence rates (IRs) for each IPD manifestation episode were calculated by dividing the total number of episodes by the total number of PY of health plan enrollment for all children in the corresponding calendar year and were expressed as episodes per 100,000 PY. The annual IRs of overall IPD were calculated as the summation of four manifestations IRs. As the exact date of birth was not available, age was imputed assuming July 1 as the birthdate within each study year. Average IRs were calculated for each of the five time periods of interest, both for the overall population and stratified by age group, for the commercially insured population and for the Medicaid population.

Nationally representative IRs were calculated via direct standardization of IRs in the MarketScan population by age, sex, and insurance type (commercial versus Medicaid). Census data for each study year were obtained from the US Census Bureau database. Estimates of the US population by sex, age, and insurance type were calculated for July 1 of each study year by applying the average proportion of individuals with private and government health insurance for the age group from 0 to 17 years across all age-sex categories. The IPD IRs in the general US pediatric population were calculated by multiplying the IRs for each age-sex-insurance type group in the MarketScan data with the proportion of that group in the general US pediatric population and summing across all groups. This approach relies on the fact that Medicaid and private insurance account for over 95% of coverage for children in the US during the study period, and uninsured children are a very small proportion of the US pediatric population.

#### Analyses of time trends

Changes in the temporal trends of IRs before and after the introduction of PCV7 and PCV13 were assessed using interrupted time series (ITS) analyses, among the commercially insured population. These analyses were conducted using generalized linear models with a negative binomial distribution and log link. The IPD episode counts in each month were modeled in a segmented regression framework, with the number of enrolled children in each month included as an offset term. In the ITS analyses of CCAE data, the monthly IRs of IPD in each vaccine period (early PCV7, late PCV7, early PCV13, late PCV13) were compared to the previous period, and monthly indicators were used to adjust for seasonal fluctuations in IRs in the models. In the analyses of Medicaid databases, the early and late PCV13 periods were compared to the prior period, respectively, and seasonal fluctuations were adjusted for in the same way as in the CCAE data analyses.

Incidence rate ratios (IRRs) and 95% confidence intervals (95% CIs) were estimated for change in levels (immediate change in the IRs compared with the previous period) and changes in trend (a gradual monthly change in the current IRs over time compared to the trends in the previous period) for each period. Predicted IRs were also obtained from the negative binomial models and linear changes in trends associated with each PCV vaccine period were assessed. ITS analyses were conducted for children overall, and separately by age groups (< 2, 2–4, and 5–17 years). Statistical analyses were conducted using SAS version 9.4 (SAS Institute, Inc., Cary, North Carolina) and R statistical software (R Foundation for Statistical Computing, Vienna, Austria). Additional details on disease episode definition, estimation of national incidence rates, and ITS model specification are included in the Supplemental Appendix.

## Results

### Demographic characteristics and risk factors

Over the course of the study period, an average of 7.08 million commercially insured children under 18 years of age contributed 5.81 million PY at risk each year. In addition, 4.27 million children under the age of 18 years who were enrolled in Medicaid plans contributed 3.49 million PY at risk on average each year. The sample sizes for the populations at risk are shown in Supplemental Table A2 (commercially insured) and Supplemental Table A3 (Medicaid population).

Demographic characteristics for the commercially insured and Medicaid populations at risk are shown in Supplemental Table A4 for each PCV period. Demographic characteristics for the commercially insured population with IPD during each PCV period are described in Table 1. Slightly more than half of the commercially insured patients with IPD were male, and the proportion under 2 years of age ranged from 58.1% in the pre-PCV7 period to 37.1% in the late PCV13 period. Most of these patients lived in urban areas, ranging from 65.4% in the pre-PCV7 to 84.8% in the late PCV13 periods, and in the South (ranging from 35.9% to 48.7% across study periods). Demographic characteristics for the Medicaid population with IPD were similar to the commercially insured patients with IPD (though the distributions of health plan types were quite different), and are shown in Supplemental Table A5.

**Table 1:** Demographic characteristics of commercially insured children aged < 18 years with IPD episodes, by PCV period (1998–2018)

	**Pre-PCV7**	**Early PCV7**	**Late PCV7**	**Early PCV13**	**Late PCV13**
	**(1998–1999)**	**(2001–2005)**	**(2006–2009)**	**(2011–2013)**	**(2014–2018)**
**Total number of patients**^**a**^**,** **N**	**136**	**734**	**1,409**	**871**	**796**
**Age, mean (SD)**^**b,c,d**^	2.84 (4.00)	4.21 (5.14)	4.13 (5.14)	5.18 (5.42)	5.15 (5.38)
< 2 years, %	58.1	46.3	46.1	37.8	37.1
2–4 years, %	20.6	20.3	20.3	17.8	19.8
5–17 years, %	21.3	33.4	33.6	44.4	43.1
**Male, %**	52.2	53.7	54.0	58.0	54.8
**Region**					
Northeast, %	16.2	10.2	9.4	16.9	17.1
North Central, %	30.1	21.4	22.4	23.2	21.2
South, %	41.9	44.4	48.7	35.9	41.3
West, %	4.4	22.8	18.9	20.3	19.2
Missing/unknown, %	7.4	1.2	0.6	3.7	1.1
**Urbanicity**					
Urban, %	65.4	78.7	80.6	79.2	84.8
Rural, %	27.2	20.0	18.8	17.1	11.6
Missing, %	7.4	1.2	0.6	3.7	3.6
**Health plan types**					
HMO/EPO, %	9.6	22.2	16.9	16.3	9.0
PPO/POS, %	52.9	68.8	74.8	68.9	65.2
HDHP/CDHP, %	0.0	2.0	2.2	6.9	19.1
FFS, %	37.5	4.9	2.1	1.0	1.8
Missing, %	0.0	2.0	4.0	6.9	4.9

*Abbreviations*: *CDHP* Consumer directed health plan, *EPO* Exclusive provider organization, *FFS* Fee-for-service, *HDHP* High-deductible health plan, *HMO* Health maintenance organization, *IPD* Invasive pneumococcal disease, *PCV* Pneumococcal conjugate vaccine, *POS* Point of service, *PPO* Preferred provider organization, *SD* Standard deviation

^a^Patients' demographic characteristics and risk factors were first determined by each calendar year and then combined by PCV periods, assuming each year has distinct patient population

^b^Patients' month and day of birth was imputed as July 1st for all patients. Age at onset was calculated as the difference between condition start date and imputed birth date. Patients with negative age at onset were included in the analysis

^c^For each calendar year, patients' demographic characteristics were determined at the index episode, which was defined as the first IPD episode in the given calendar year

^d^Standard deviations for age in each vaccine period were calculated using the pooled standard deviation of the samples in relevant years

Risk factors for pneumococcal disease among children enrolled in commercial insurance and Medicaid plans are described in Supplemental Table A6. The percentage of children with IPD who had any underlying risk factor increased steadily over time, from 14.3% in the pre-PCV7 period to 44.6% in the late PCV13 period in commercially insured patients and from 31.1% in the early PCV7 period to 56.0% in the late PCV13 period for patients enrolled in Medicaid plans. Among commercially insured children with IPD, the prevalence of chronic lung disease, including asthma, increased from 6.1% in the pre-PCV7 period to 15.9% in the late PCV period, and the prevalence of cancer and iatrogenic immunosuppression increased from 5.1% to 14.6% over the same time period. The prevalence of congenital or acquired immunodeficiency, chronic heart disease, solid organ transplant, and sickle cell disease or other hemoglobinopathies and anatomic or functional asplenia, increased from ≤ 1.0% in the pre-PCV7 period to 17.3%, 8.9%, 7.7%, and 5.2%, respectively, during the late PCV13 period. These 6 risk factors were also the most prevalent among children with IPD enrolled in Medicaid plans, but the prevalence was higher in the pre-PCV7 period. From the pre-PCV7 to the late PCV13 period, the prevalence of chronic lung disease including asthma increased from 14.9% to 22.3%, cancer and iatrogenic suppression increased from 8.0% to 21.3%, congenital or acquired immunodeficiency from 5.6% to 19.6%, chronic heart disease from 6.8% to 12.1%, sickle cell disease or other hemoglobinopathies and anatomic or functional asplenia from 8.2% to 9.6%, and solid organ transplant from 4.8% to 7.3%.

### Crude incidence rates of IPD

#### Overall IPD episodes

**Fig. 1:**
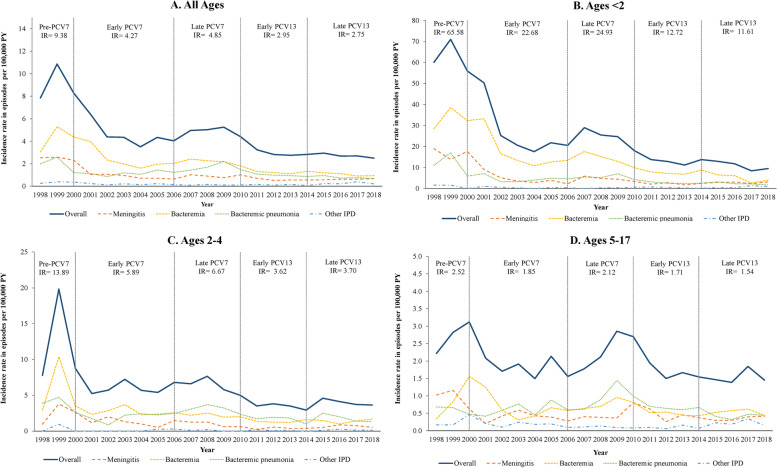
Trends in annual incidence rates of IPD by age group among commercially insured children aged < 18 years, in episodes per 100,000 person-years (1998–2018). Abbreviations: IPD: invasive pneumococcal disease; IR: incidence rate; PCV: pneumococcal conjugate vaccine, PY: person-years. Note: Average IRs for overall pneumococcal-specific IPD episodes are shown for each of the PCV periods

Annual IRs for IPD episodes among commercially insured children are presented graphically in time series plots, overall and by age groups (Fig. 1, solid lines). In the commercially insured population, IPD IRs decreased over the course of the study, from 9.4 episodes/100,000 PY in the pre-PCV7 period to 2.8 episodes/100,000 PY in the late PCV13 period (Fig. 1A). IRs were highest in the subgroup of children under 2 years of age, and decreased from 65.6 to 11.6 episodes per 100,000 PY from the pre-PCV7 period to the late PCV13 period (Fig. 1B). IRs decreased among children aged 2–4 years from 13.9 to 3.7 episodes per 100,000 PY from the pre-PCV7 period to the late PCV13 period (Fig. 1C). IRs were lowest among patients aged 5–17 years; these rates fluctuated over the course of the study with a slightly decreased trend from 2.5 to 1.5 episodes per 100,000 PY from the pre-PCV7 period to the late PCV13 period (Fig. 1D). Overall, the decrease in IPD rates was substantial during 1999 and 2002 (from the pre-PCV7 to the early PC7 period). However, an increase in IPD rates was observed during 2006 and 2008 (the late PCV7 period).

Changes in annual IRs for IPD episodes were similar among children enrolled in Medicaid plans, with the exception of a temporary spike in IR in the early PCV7 period (see Supplemental Figure A1 and Supplemental Table A7). IPD IRs decreased from 11.3 to 4.2 episodes/100,000 PY between the early PCV7 (2001–2005) and the late PCV13 period. The IPD IRs were highest in children under 2 years of age, decreasing from 42.6 to 12.8 episodes/100,000 PY from the early PCV7 to the late PCV13 period. IRs also decreased among children aged 2–4 years and aged 5–17 years over this time period, from 9.8 to 5.6 episodes, and 3.8 to 2.3 episodes/100,000 PY, respectively. IPD rates decreased substantially during the early-PCV7 period, increased during the late PCV7 period, and then trended downwards throughout the early and late PCV13 periods.

#### Episodes of meningitis, bacteremia, bacteremic pneumonia, and other invasive pneumococcal disease

Annual IRs for episodes of pneumococcal-specific meningitis, bacteremia, bacteremic pneumonia, and other IPD among commercially insured children are presented in Fig. 1 (dashed lines) and Table 2. In general, the trend of IRs for these manifestations closely follows the patterns of overall IPD episodes. The IR for meningitis decreased over the course of the study from 2.6 per 100,000 PY in the pre-PCV period to 0.6 episodes per 100,000 PY in the late PCV13 period (Table 2). The most substantial decrease was observed among children under 2 years of age. Bacteremia is the most common condition among all IPD manifestations. The IR for bacteremia decreased from 4.2 to 1.1 episodes per 100,000 PY from the pre-PCV7 to the late PCV13 period (Table 2). The IR for bacteremic pneumonia decreased from 2.3 to 0.8 episodes per 100,000 PY over this period (Table 2). Other IPDs (e.g., arthritis, peritonitis, pericarditis, endocarditis, and osteomyelitis) were the least common IPD manifestation, with the IR ranging from 0.3 to 0.1 episodes per 100,000 PY. The IR for other IPDs fluctuated over the course of the study with a slightly decreased trend, both among the overall commercial issued population, and in each age group.

**Table 2:** Incidence rates and 95% confidence intervals of pneumococcal-specific meningitis, bacteremia, bacteremic pneumonia, and other IPD, among commercially insured children aged < 18 years, in episodes per 100,000 person-years, by PCV period (1998–2018)^a,b^

	**All ages**					**Ages < 2**				
**Period**	**Overall IPD**	**Meningitis**	**Bacteremia**	**Bacteremic** **pneumonia**	**Other IPD**	**Overall IPD**	**Meningitis**	**Bacteremia**	**Bacteremic** **pneumonia**	**Other IPD**
	**IR** **(95% CI)**	**IR** **(95% CI)**	**IR** **(95% CI)**	**IR** **(95% CI)**	**IR** **(95% CI)**	**IR** **(95% CI)**	**IR** **(95% CI)**	**IR** **(95% CI)**	**IR** **(95% CI)**	**IR** **(95% CI)**
Pre-PCV7	9.38 (7.96; 11.04)	2.56 (1.87; 3.50)	4.20 (3.29; 5.36)	2.29 (1.65; 3.19)	0.33 (0.14; 0.77)	65.58 (52.97; 81.18)	16.39 (10.72; 25.06)	33.57 (24.92; 45.21)	14.05 (8.89; 22.21)	1.56 (0.43; 5.69)
Early PCV7	4.27 (3.97; 4.58)	0.84 (0.71; 0.99)	2.06 (1.86; 2.28)	1.18 (1.03; 1.35)	0.19 (0.13; 0.26)	22.68 (20.43; 25.18)	4.01 (3.13; 5.14)	14.15 (12.40; 16.15)	4.20 (3.30; 5.35)	0.32 (0.14; 0.76)
Late PCV7	4.85 (4.61; 5.10)	0.83 (0.73; 0.94)	2.22 (2.06; 2.40)	1.67 (1.53; 1.82)	0.13 (0.09; 0.17)	24.93 (23.13; 26.87)	4.32 (3.61; 5.17)	14.72 (13.35; 16.23)	5.64 (4.81; 6.60)	0.26 (0.12; 0.53)
Early PCV13	2.95 (2.77; 3.15)	0.59 (0.51; 0.69)	1.21 (1.10; 1.34)	1.00 (0.90; 1.12)	0.14 (0.10; 0.19)	12.72 (11.44; 14.14)	2.19 (1.70; 2.83)	7.33 (6.37; 8.42)	2.68 (2.13; 3.37)	0.52 (0.31; 0.87)
Late PCV13	2.75 (2.57; 2.94)	0.60 (0.52; 0.69)	1.13 (1.02; 1.26)	0.81 (0.72; 0.92)	0.20 (0.16; 0.26)	11.61 (10.41; 12.94)	2.72 (2.18; 3.41)	5.98 (5.14; 6.96)	2.36 (1.86; 3.01)	0.54 (0.33; 0.89)
	**Ages 2–4**					**Ages 5–17**				
**Period**	**Overall IPD**	**Meningitis**	**Bacteremia**	**Bacteremic** **pneumonia**	**Other IPD**	**Overall IPD**	**Meningitis**	**Bacteremia**	**Bacteremic** **pneumonia**	**Other IPD**
	**IR** **(95% CI)**	**IR** **(95% CI)**	**IR** **(95% CI)**	**IR** **(95% CI)**	**IR** **(95% CI)**	**IR** **(95% CI)**	**IR** **(95% CI)**	**IR** **(95% CI)**	**IR** **(95% CI)**	**IR** **(95% CI)**
Pre-PCV7	13.89 (9.67; 19.95)	2.40 (1.02; 5.61)	6.71 (3.99; 11.26)	4.31 (2.27; 8.19)	0.48 (0.08; 2.71)	2.52 (1.77; 3.60)	1.09 (0.64; 1.87)	0.59 (0.29; 1.22)	0.67 (0.34; 1.33)	0.17 (0.05; 0.61)
Early PCV7	5.89 (5.03; 6.91)	1.05 (0.72; 1.53)	2.69 (2.13; 3.41)	2.07 (1.58; 2.71)	0.08 (0.02; 0.28)	1.85 (1.63; 2.09)	0.44 (0.34; 0.56)	0.55 (0.44; 0.70)	0.67 (0.54; 0.82)	0.19 (0.13; 0.28)
Late PCV7	6.67 (5.96; 7.47)	1.11 (0.84; 1.47)	2.29 (1.89; 2.78)	3.14 (2.66; 3.70)	0.13 (0.06; 0.29)	2.12 (1.94; 2.31)	0.36 (0.29; 0.45)	0.73 (0.63; 0.85)	0.92 (0.80; 1.05)	0.11 (0.07; 0.16)
Early PCV13	3.62 (3.10; 4.22)	0.40 (0.25; 0.63)	1.28 (0.99; 1.65)	1.83 (1.48; 2.27)	0.11 (0.05; 0.26)	1.71 (1.55; 1.88)	0.45 (0.37; 0.54)	0.50 (0.42; 0.60)	0.65 (0.56; 0.76)	0.10 (0.07; 0.15)
Late PCV13	3.70 (3.18; 4.31)	0.58 (0.40; 0.85)	1.45 (1.14; 1.85)	1.56 (1.24; 1.97)	0.11 (0.05; 0.26)	1.54 (1.39; 1.70)	0.36 (0.29; 0.44)	0.51 (0.43; 0.61)	0.49 (0.41; 0.59)	0.18 (0.13; 0.24)

*Abbreviations*: *CI* Confidence interval, *IPD* Invasive pneumococcal disease, *IR* Incidence rate, *PCV* Pneumococcal conjugate vaccine

^a^Confidence intervals were calculated using Pearson method

^b^Time periods are defined as follows: Pre-PCV7: 1998–1999; Early PCV7: 2001–2005; Late PCV7: 2006–2009; Early PCV13: 2011–2013; Late PCV13: 2014–2018. Years 2000 and 2010 are considered transition years and were excluded

The annual IRs for pneumococcal-specific episodes of meningitis, bacteremia, bacteremic pneumonia, and other IPD for children enrolled in Medicaid plans are presented in Supplemental Appendix (Fig. A1, dashed lines, and Supplemental Table A7). The trend of the annual IRs was similar to that among the commercially insured children; however, IRs were in general slightly higher among children enrolled in Medicaid plans throughout the study period. In the late PCV13 period, the annual IRs for meningitis, bacteremia and bacteremic pneumonia in Medicaid-insured children all ages were 0.89, 1.88, 1.23, and 0.17 episodes/100,000 PY, respectively, higher than the rates in the commercially insured population.

### National incidence rate estimates

**Fig. 2:**
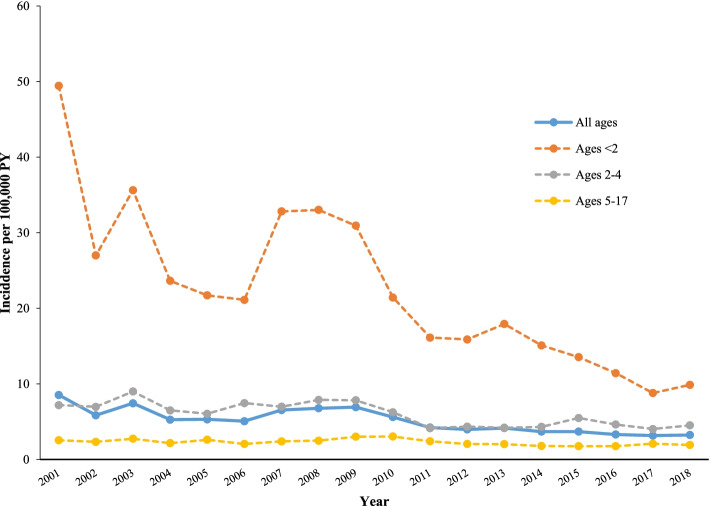
National estimates of annual IPD incidence rates among US children aged < 18 Years (2001–2018). Abbreviations: IPD: invasive pneumococcal disease; PY: person-years. Note: Incidence rates were adjusted using data from the U.S. Census Bureau, Current Population Survey and Annual Social and Economic Supplements. Census data for each study year were obtained from the US Census Bureau database. Estimates of the July 1st US population by sex, age, and insurance type were calculated for each study year by applying the average proportion of individuals with private and government health insurance for the 0–17 age group across all age-sex categories. IPD incidence rates in the general US pediatric population were calculated by multiplying the incidence rates for each age-sex-insurance type group in the MarketScan data with the proportion of that group in the general US pediatric population, and summing across all groups

The estimates for nationally representative annual IRs for IPD are presented graphically in Fig. 2, for the overall pediatric population in the US, and by age group. Nationally representative annual IRs for each IPD manifestation (meningitis, bacteremia, and bacteremic pneumonia) are shown in Table 3. The IR of IPD for children of all ages decreased over the course of the study, from 8.5 episodes per 100,000 PY in 2001 to 3.2 episodes per 100,000 PY in 2018. Despite the overall decrease in IRs for all age groups, fluctuations in IRs were observed over the course of the study, and IRs reached a peak around 2009 in all children (excluding the Year 2001, which had the highest IR). The trends for meningitis, bacteremia, and bacteremic pneumonia were similar to the pattern for overall IPD episodes. The IR for meningitis, bacteremia, and bacteremic pneumonia decreased from 1.5, 5.1, and 1.7 per 100,000 PY in 2001 to 0.9, 1.2, and 1.0, respectively, in 2018. The nationally representative annual IR of other IPD was not estimated due the low incidence throughout the study period.

**Table 3:** National estimates of annual incidence rates of IPD and its manifestations among US children aged < 18 Years, in episodes per 100,000 person-years (2001–2018)

Year	Overall IPD				Meningitis				Bacteremia				Bacteremic pneumonia			
	**All ages**	**Ages** ** < 2**	**Ages** **2–4**	**Ages** **5–17**	**All ages**	**Ages** ** < 2**	**Ages** **2–4**	**Ages** **5–17**	**All ages**	**Ages** ** < 2**	**Ages** **2–4**	**Ages** **5–17**	**All ages**	**Ages** ** < 2**	**Ages** **2–4**	**Ages** **5–17**
2001	8.53	49.40	7.19	2.54	1.53	9.56	1.32	0.34	5.09	30.90	3.82	1.41	1.65	7.95	1.94	0.61
2002	5.84	27.00	6.96	2.33	1.30	5.20	1.61	0.63	3.16	17.20	3.74	0.86	1.19	4.06	1.55	0.66
2003	7.43	35.60	8.99	2.74	1.01	3.73	1.24	0.54	4.29	26.00	5.09	0.76	1.78	5.48	2.59	1.03
2004	5.26	23.60	6.49	2.16	0.92	3.29	0.93	0.55	2.62	15.40	2.93	0.58	1.49	4.95	2.63	0.69
2005	5.30	21.70	6.05	2.60	0.87	3.99	0.52	0.47	2.23	11.30	2.48	0.77	1.91	5.96	2.81	1.09
2006	5.06	21.10	7.45	2.04	0.77	2.18	1.37	0.41	2.44	13.00	2.70	0.76	1.66	5.61	3.12	0.72
2007	6.54	32.80	6.98	2.39	1.12	5.09	1.12	0.51	3.40	20.80	2.72	0.88	1.83	6.42	3.08	0.84
2008	6.77	33.00	7.88	2.48	1.13	5.29	1.22	0.47	3.40	21.20	2.82	0.80	2.06	6.17	3.65	1.06
2009	6.91	30.90	7.82	3.01	0.84	4.25	0.62	0.37	3.06	17.10	3.01	0.90	2.75	9.10	4.13	1.45
2010	5.61	21.40	6.25	3.03	0.99	3.41	0.62	0.71	2.43	11.80	2.45	0.99	2.01	5.74	3.05	1.20
2011	4.22	16.10	4.14	2.40	0.73	2.18	0.51	0.56	1.82	9.21	1.87	0.67	1.47	4.04	1.64	1.04
2012	3.97	15.90	4.35	2.05	0.74	3.06	0.65	0.41	1.75	8.41	1.68	0.74	1.34	3.87	1.93	0.82
2013	4.15	17.90	4.19	2.03	0.66	2.06	0.56	0.47	2.17	12.80	1.86	0.61	1.16	2.70	1.68	0.81
2014	3.68	15.10	4.31	1.78	0.67	2.07	0.82	0.41	1.84	9.78	1.88	0.61	1.06	2.87	1.62	0.66
2015	3.69	13.50	5.48	1.76	0.68	2.59	0.66	0.40	1.61	7.24	2.05	0.65	1.19	3.31	2.70	0.52
2016	3.30	11.40	4.62	1.75	0.82	2.81	1.01	0.46	1.37	5.78	1.50	0.66	0.90	2.23	1.88	0.46
2017	3.15	8.79	4.03	2.08	0.74	2.11	0.85	0.50	1.21	3.41	1.72	0.75	0.91	2.23	1.40	0.59
2018	3.23	9.87	4.51	1.92	0.85	2.77	0.91	0.54	1.21	3.92	1.95	0.62	0.98	2.56	1.58	0.60

*Abbreviations*: *IPD* Invasive pneumococcal disease, *PY* Person-years

Note: Incidence rates were adjusted using data from the U.S. Census Bureau, Current Population Survey and Annual Social and Economic Supplements. Census data for each study year were obtained from the US Census Bureau database. Estimates of the July 1st US population by sex, age, and insurance type were calculated for each study year by applying the average proportion of individuals with private and government health insurance for the 0–17 age group across all age-sex categories. IPD incidence rates in the general US pediatric population were calculated by multiplying the incidence rates for each age-sex-insurance type group in the MarketScan data with the proportion of that group in the general US pediatric population, and summing across all groups

### Interrupted time series results

The estimated IRRs from the ITS analyses in the commercially insured children are shown in Table 4, and the monthly IRs of IPD episodes, along with fitted linear trends for each period, are shown in Supplemental Figure A2. Estimated IRRs, and monthly IRs of IPD episodes along with fitted linear trends from the ITS analyses in the Medicaid population are shown in Supplemental Table A8 and Supplemental Figure A3, respectively. Results discussed in the text for specific age groups in Table 4 and Supplemental Table A8 are statistically significant (*p*-value < 0.05).

**Table 4:** Estimates from ITS analyses of monthly unspecified IPD episode incidence rates in commercially insured children aged < 18 years (1998–2018)

		**All ages**		**Ages < 2**		**Ages 2–4**		**Ages 5–17**	
**Period**^**a**^	**IRR**	**IRR** **(95% CI)**^**b,**^^**c**^	***p*-value**	**IRR** **(95% CI)**^**b,**^^**c**^	***p*-value**	**IRR** **(95% CI)**	***p*-value**	**IRR** **(95% CI)**^**b,**^^**c**^	***p*-value**
Pre-PCV7	Base Trend	1.022 (1.008; 1.036)	0.002**	1.013 (0.985; 1.043)	0.368	1.071 (1.016; 1.129)	0.011*	1.000 (0.949; 1.054)	0.994
Early PCV7	Change in Level	0.500 (0.333; 0.750)	0.001**	0.607 (0.318; 1.159)	0.13	0.232 (0.117; 0.461)	0.001***	0.735 (0.280; 1.932)	0.533
	Change in Trend	0.972 (0.958; 0.986)	0.001***	0.972 (0.943; 1.003)	0.076	0.933 (0.885; 0.983)	0.010*	1.000 (0.949; 1.054)	0.995
Late PCV7	Change in Level	1.123 (0.906; 1.391)	0.291	1.480 (1.036; 2.115)	0.031*	1.193 (0.877; 1.622)	0.262	0.720 (0.495; 1.047)	0.085
	Change in Trend	1.014 (1.006; 1.021)	0.001**	1.017 (1.006; 1.029)	0.003**	1.000 (0.990; 1.011)	0.943	1.017 (1.005; 1.030)	0.005**
Early PCV13	Change in Level	0.653 (0.522; 0.817)	0.001***	0.637 (0.476; 0.851)	0.002**	0.669 (0.364; 1.231)	0.197	0.653 (0.449; 0.951)	0.026*
	Change in Trend	0.986 (0.980; 0.993)	0.001***	0.988 (0.979; 0.997)	0.009**	0.994 (0.977; 1.012)	0.538	0.978 (0.965; 0.991)	0.001**
Late PCV13	Change in Level	1.141 (0.921; 1.415)	0.228	1.390 (1.065; 1.815)	0.016*	1.064 (0.692; 1.634)	0.778	0.964 (0.693; 1.340)	0.826
	Change in Trend	1.005 (0.998; 1.012)	0.17	1.001 (0.990; 1.011)	0.886	1.010 (0.991; 1.028)	0.312	1.006 (0.994; 1.018)	0.331

*Abbreviations*: *CI* Confidence internal, *IPD* Invasive pneumococcal disease, *IRR* Incidence rate ratio, *ITS* Interrupted time series, *PCV* Pneumococcal conjugate vaccine

Significance codes: ∗ *p* < 0.05; ∗  ∗ *p* < 0.01; ∗  ∗  ∗ *p* < 0.001. *P*-values less than 0.001 are shown as 0.001***

^a^Time periods are defined as follows: Pre PCV7: 1998–1999; Early PCV7: 2001–2005; Late PCV7: 2006–2009; Early PCV13: 2011–2013; Late PCV13: 2014–2018. Years 2000 and 2010 are considered transition years and were excluded from the model

^b^All coefficients were obtained through a negative binomial model with a log link, controlling for seasonality using monthly indicators. IRRs represent the exponentiated regression coefficients and indicate a multiplicative change. Model intercepts are not shown

^c^Confidence intervals have been adjusted for heteroscedasticity

In commercially insured children under 2 years of age, there was a 48.0% immediate increase (IRR: 1.480; 95% CI: 1.036, 2.155; *p* = 0.031) and a 1.7% per month gradual increase (IRR: 1.017; 95% CI: 1.006, 1.029; *p* = 0.003) in monthly IRs in the late PCV7 period compared to early PCV7 period. In the early PCV13 period, there was a 36.3% immediate decrease (IRR: 0.637; 95% CI: 0.476, 0.851; *p* = 0.002) and a 1.2% per month gradual decrease (IRR: 0.988; 95% CI: 0.979, 0.997; *p* = 0.009) in monthly IRs compared to the late PCV7 period. In the late PCV13 period, there was a 39.0% immediate increase (IRR: 1.390; 95% CI: 1.065, 1.815; *p* = 0.016) compared to the early PCV13 period. In children in this age group enrolled in Medicaid plans, there was a gradual increase in monthly IRs of 1.5% (IRR: 1.015; 95% CI: 1.006, 1.023; *p* = 0.001) in the late PCV7 period. There was a 70.4% immediate decrease (IRR: 0.296; 95% CI: 0.197, 0.446; *p* = 0.001) in the early PCV13 period. In the late PCV13 period, there was a 38.1% immediate decrease (IRR: 0.619; 95% CI: 0.432, 0.888; *p* = 0.009) and a 2.4% per month gradual decrease (IRR: 0.976; 95% CI: 0.963, 0.990; *p* = 0.001).

In commercially insured children from 2 to 4 years of age, there was a gradual increase in monthly IRs of 7.1% per month (IRR: 1.071; 95% CI: 1.016, 1.129; *p* = 0.011) during the pre-PCV7 period. Compared to this period, there was a 76.8% immediate decrease (IRR: 0.232; 95% CI: 0.117, 0.461; *p* = 0.001) and a 6.7% per month gradual decrease (IRR: 0.933; 95% CI: 0.885, 0.983; *p* = 0.010) in monthly IRs in the early PCV7 period. In children from 5 to 17 years of age, there was a 1.7% gradual increase (IRR: 1.017; 95% CI: 1.005, 1.030; *p* = 0.005) in monthly IRs in the late PCV7 period, compared to the early PCV7 period. In the early PCV13 period, there was a 34.7% immediate decrease (IRR: 0.653; 95% CI: 0.449, 0.951; *p* = 0.026) and a 2.2% per month gradual decrease (IRR: 0.978; 95% CI: 0.965, 0.991; *p* = 0.001) in monthly IRs compared to late PCV7 period. In children in these two age groups enrolled in Medicaid plans, no significant immediate or gradual changes were observed from the late PCV7 to the late PCV13 periods.

## Discussion

In this study of administrative claims data, we found that the IRs of overall IPD episodes, pneumococcal meningitis, pneumococcal bacteremia, and pneumococcal bacteremic pneumonia decreased over time, both among children with commercial insurance and children in Medicaid plans. While substantial reductions were observed overall, and for children under 2 years and from 2 to 4 years of age, a smaller reduction was found in children from 5 to 17 years of age. Furthermore, in the late PCV13 period, IPD IRs were generally slightly higher in the Medicaid population, compared with the commercially insured population, across all age groups, both for overall IPD episodes, and for meningitis, bacteremia and bacteremic pneumonia.

The crude IRs and ITS analyses indicate that while the pre-PCV7 period (for the commercially insured) and the late PCV7 period (for the commercially insured and Medicaid) were associated with an increase in IPD IRs, there was a substantial reduction in the IR of IPD episodes over time, particularly during the early PCV7 and early PCV13 periods (for the commercially insured) and during the early PCV13 period (for Medicaid). The increase in IPD rates during the late PCV7 period (particularly from years 2006 to 2008), has previously been attributed to serotype replacement, with IPD caused by non-vaccine serotypes. Indeed, a report based on the ABCs data showed an increase in IPD cases due to non-PCV7 serotypes in the year 2007 [34]. A prior systemic review study also found an increase in non-vaccine serotypes after pneumococcal vaccination, which indicates serotype replacement in IPD [13].

The estimates for nationally representative IRs indicate that for all three age groups examined, the IRs of IPD decreased by about 62% at the national level over the course of the study, with the highest reduction, both in absolute and relative terms, occurring in children aged under 2 years of age. However, there is still a substantial residual burden of IPD in children nationally (3.2 episodes per 100,000). Among all IPD manifestations, the highest reduction, both in absolute and relative terms, occurred in the IRs for bacteremia, which decreased overall by nearly 76.2% overall between 2001 and 2018, with the most substantial reduction in children aged < 2 years (87.3%).

This study found that the percentage of children with IPD with any underlying risk factor increased over time, to about 50% in the late PCV13 period, with higher rates for children enrolled in Medicaid plans. The most common underlying conditions included chronic lung disease, cancer and iatrogenic immunosuppression, congenital or acquired immunodeficiency, chronic heart disease, solid organ transplant, and sickle cell disease or other hemoglobinopathies and anatomic or functional asplenia. A similar finding was noted in a recent study of IPD in US children’s hospitals, which found that nearly half of children with IPD in the late PCV13 period had underlying conditions. Non-PCV13 serotypes accounted for about three-quarters of IPD episodes, and an underlying condition was significantly more common in cases with IPD due to non-vaccine serotypes; other studies have reported similar findings as well [26, 35]. The 23-valent polysaccharide vaccine (PPSV23) contains additional serotypes, and is recommended for children with select underlying conditions, but is not immunogenic in children under 2 years of age. Newer conjugate vaccines with coverage against additional serotypes, which are currently undergoing evaluation in children, may provide greater protection in this population [27–31]. However, continued surveillance and additional data will be necessary to demonstrate vaccine effectiveness, especially among children with underlying conditions.

Despite differences in study methodology and data sources which may render cross-study comparisons challenging, our results are generally consistent with a number of previously published studies [24]. The observed IRs for IPD in this analysis are consistent with, although slightly lower than, the ABCs report for the period from 1998 to 2018, and with other published reports as well [14, 19, 36, 37]. Consistent with previous reports, this analysis indicates that the IR of IPD has declined dramatically since the universal administration of PCVs was implemented in the year 2000, with the most significant decline occurred during early the PCV7 period and early PCV13 period [3, 19, 26, 37]. An recent study describing the 20-year impact of PCVs also found substantial declines, particularly during the early PCV7 period [22].

Consistent with previously published reports, this study found that the IR of IPD varies by age groups, with highest rates seen among younger children [38–40]. Bacteremia is the most common manifestation of IPD, especially among children aged < 2 years [41]. This study found that the most significant declines in IRs for IPD after the PCVs became available occurred in infants and young children, the pediatric age group with the highest risk of IPD. This finding was also reported in other studies which have evaluated the IRs of IPD by age group since the introduction of PCV [22, 23].

To our knowledge, this is the first study to comprehensively investigate the IRs of overall and specific manifestations of IPDs from 1998 to 2018, before and after the introduction of PCV7 and PCV13 among children in the US. However, the study has several limitations. First, as with any large claims database, miscoding of diagnoses may occur, potentially leading to misclassification and measurement error. Pathogen-specific disease episodes caused by *S. pneumoniae* were identified using diagnosis codes; however, bacterial culture and specific serotypes results were not available. Therefore, IRs of IPD and its specific manifestations that are estimated from claims database are likely different from the IRs generated from surveillance reports. In addition, the lack of data on serotype limits the ability to determine the potential impact of higher valent vaccines in further reducing IPD burden. Second, the change from ICD-9 to ICD-10 diagnosis code systems in 2015 may have affected the classification of diseases over time, potentially affecting the comparison of periods before versus after this transition. Finally, although the MarketScan database is considered representative of commercial health plans in the US, it is based on non-random sampling of employer-sponsored health plans, with large employers over-represented. Therefore, the national estimates of IPD IRs may not be generalizable to children with other types of commercial insurance. In addition, the Medicaid databases only include data from a convenience sample of states, and data are not available for children without health insurance coverage. Therefore, the national IR estimates may not be fully representative of the pediatric population in the US.

## Conclusions

Incidence rates of IPD decreased dramatically in both commercially and Medicaid insured children from 1998 to 2018. Substantial IPD declines were found during the early PCV7 and early PCV13 periods. Similarly, the incidence rates of meningitis, bacteremia, and bacteremic pneumonia decreased consistently during this period. The residual burden of invasive pneumococcal disease remains substantial, particularly in children < 2 years of age. The extent to which future PCVs will impact pneumococcal disease will depend on the composition of vaccine-type serotypes, and the immune response that can be generated among children.

## Supplementary Information

**Additional file 1:** **Supplemental Table A1.** Diagnosis and procedure codes used in the study. **Supplemental Table A2.** Size of MarketScan commercially insured children population at risk in person-years and estimates of the the total US pediatric population with commercial insurance (1998-2018). **Supplemental Table A3.** Size of MarketScan Medicaid-insured children population at risk in person-years and estimates of the the total US pediatric population with Medicaid coverage (2001-2018). **Supplemental Table A4.** Demographic characteristics of the population at risk (1998-2018). **Supplemental Table A5.** Demographic characteristics of Medicaid insured children aged < 18 years with IPD episodes, by PCV period (2001-2018). **Supplemental Table A6.** Presence of risk factors for pneumococcal disease among IPD patients aged <18 years in the 6 months prior to IPD episodes, 1998-2018. **Supplemental Table A7.** Incidence rates and 95% confidence intervals of unspecified meningitis, bacteremia, bacteremic pneumonia, and other IPD, among Medicaid insured children, in episodes per 100,000 PY (2001-2018). **Supplemental Table A8.** Estimates from ITS analysis of monthly IPD episode IRs for the Medicaid-insured children aged <18 years (2006-2018). **Supplemental Figure A1.** Annual incidence rates of IPD episodes in Medicaid insured children by age group, in episodes per 100,000 PY (2001-2018). **Supplemental Figure A2.** Monthly incidence rates and linear time trends predicted from the ITS models in the commercially insured population aged <18 years, in episodes per 100,000 PY (1998-2018). **Supplemental Figure A3.** Monthly incidence rates and linear time trends predicted from the ITS models in the Medicaid-insured population aged <18 years, in episodes per 100,000 PY (2006-2018). **Supplemental Figure A4.** ACF and PACF of the residuals from the ITS model of IPD among commercially insured children aged 0-17 years (1998-2018). **Supplemental Figure A5.** ACF and PACF of the residuals from the ITS model of IPD among Medicaid insured children aged 0-17 years (2006-2018).

## Data Availability

The data that support the findings of this study are available from the IBM® MarketScan® Research Databases but restrictions apply to the availability of these data, which were used under license for the current study, and so are not publicly available. Data are however available from the authors upon reasonable request from the corresponding author with permission from IBM ® Watson Health™.

## Abbreviations

ABCs: Active Bacterial Core surveillance
ACIP: Advisory Committee on Immunization Practices
CCAE: Commercial Claims and Encounters
CIs: Confidence intervals
ICD-9/10-CM: International Classification of Diseases 9/10^th^ Revision, Clinical Modification
IPD: Invasive pneumococcal disease
IR: Incidence rate
IRRs: Incidence rate ratios
ITS: Interrupted time series
PCV: Pneumococcal conjugate vaccine
PCV13: 13-Valent PCV
PCV7: 7-Valent PCV
PPSV23: 23-Valent polysaccharide vaccine
PYs: Person-years
US: United States
