# Supplementary material for: Incidence of invasive pneumococcal disease in children with commercial insurance or Medicaid coverage in the United States before and after the introduction of 7- and 13-valent pneumococcal conjugate vaccines during 1998–2018
Source: BMC Public Health. 2022 Sep 5;22:1677. doi: 10.1186/s12889-022-14051-6 (PMC9442936; doi:10.1186/s12889-022-14051-6)

# Supplemental Appendix

## Supplemental Table A1. Diagnosis and procedure codes used in the study

| **Condition** | **ICD-9-CM** | | | **ICD-10-CM** | | | **Descriptions** |
| --- | --- | --- | --- | --- | --- | --- | --- |
| **IPD** | | | | | | | |
| **Meningitis** | 320.1 | | | G00.1 | | | Pneumococcal meningitis |
|  | 320.2+041.2 | | | G00.2+B95.3 | | | Streptococcal meningitis+ Pneumococcal infection |
|  | 320.9 +041.2 | | | G00.9+B95.3 | | | Bacterial meningitis, unspecified + Pneumococcal infection |
|  | 322.9 +041.2 | | | G03.9+B95.3 | | | Meningitis, unspecified + Pneumococcal infection |
| **Bacteremia** | 038.2 | | | A40.3 | | | Pneumococcal septicemia |
|  | 038.0+041.2 | | | A40.9+B95.3 | | | Streptococcal septicemia + Pneumococcal infection |
|  | 038.9+041.2 | | | A41.9+B95.3 | | | Unspecified septicemia + Pneumococcal infection |
|  | 790.7+041.2 | | | R78.81+B95.3 | | | Bacteremia + Pneumococcal infection |
| **Bacteremic Pneumonia** | 510.x+041.2 | | | J86.x+B95.3 | | | Empyema+ Pneumococcal infection |
|  | 513.0+041.2 | | | J85.1+B95.3 | | | Abscess of lung + Pneumococcal infection |
|  | 038.2 | and | One code from all-cause pneumonia | A40.3 | and | One code from all-cause pneumonia | One code from the list of pneumococcal specific septicemia and one code from the list of pneumonia |
|  | 038.0+041.2 |  |  | A40.9+B95.3 |  |  |  |
|  | 038.9+041.2 |  |  | A41.9+B95.3 |  |  |  |
|  | 790.7+041.2 |  |  | R78.81+B95.3 |  |  |  |
|  | 038.0 | and | 481 | A40.9 | and | J13 |  |
|  | 038.9 |  |  | A41.9 |  |  |  |
|  | 790.7 |  |  | R78.81 |  |  |  |
| **Other IPD** | **--** | | | M00.1x | | | Pneumococcal arthritis |
|  | 567.1 | | | K65.8+B95.3 | | | Pneumococcal peritonitis |
|  | 420.9x+041.2 | | | I30.1+B95.3 | | | Infective pericarditis + Pneumococcal infection |
|  | 421.0+041.2 | | | I33.0+B95.3 | | | Acute and subacute bacterial endocarditis + Pneumococcal infection |
|  | 421.1/421.9 + 041.2 | | | I33.9 +B95.3 | | | Acute and subacute endocarditis, unspecified+ Pneumococcal infection |
|  | 567.23+041.2 | | | K65.2+B95.3 | | | Spontaneous bacterial peritonitis+ Pneumococcal infection |
|  | 730.0x, 730.2x +041.2 | | | M86.1x/M86.2x/M86.9+B95.3 | | | Acute or unspecified osteomyelitis+ Pneumococcal infection |
|  | 711.0x/711.9x +041.2 | | | M00.0x, M00.2x, M00.8x, M00.9 + B95.3 | | | Pyogenic/unspecified arthritis+ Pneumococcal infection |
| **All-cause pneumonia** | | | | | | | |
| **All-cause pneumonia** | 480.x (480.0-480.3, 480.8, 480.9) | | | J12.x (J12.0, J12.1, J12.2, J12.3, J12.81, J12.89, J12.9) | | | Viral pneumonia |
|  | 481 | | | J13, J18.1 | | | Pneumococcal pneumonia |
|  | 482.x | | | A48.1, J14, J15.0, J15.1, J15.2x (J15.20, J15.211, J15.212, J15.29), J15.3, J15.4, J15.5, J15.6, J15.8, J15.9 | | | Other bacterial pneumonia |
|  | 483.x (483.0, 483.1, 483.8) | | | J15.7, J16.x (J16.0, J16.8) | | | Pneumonia due to other specified organism |
|  | 484.x (484.1, 484.3, 484.5-484.8) | | | A22.1, A37.X1, B25.0, B44.0, J17 | | | Pneumonia in infectious diseases classified elsewhere |
|  | 485 | | | J18.0 | | | Bronchopneumonia, organism unspecified |
|  | 486 | | | J18.2, J18.8, J18.9 | | | Pneumonia, organism unspecified |
|  | 487.0 | | | J09.X1, J10.0x (J10.00, J10.01, J10.08), J11.0x (J11.00, J11.08) | | | Influenza with pneumonia |

## **Additional information on IPD episode definition**

Only claims occurring during a patient’s enrollment period were included. Four IPD manifestations were separately identified. When diagnosis codes present on the same claim fulfilled the definitions for multiple manifestations, the higher-priority manifestation took precedence according to the following hierarchy to avoid double-counting: meningitis > bacteremia > bacteremic pneumonia > other IPD. Claims for lower-priority manifestation were excluded, in order to avoid attributing the health care encounters related to more severe diseases commonly caused by *S. pneumoniae*.

## **Derivation of national incidence rates**

Data for each study year were obtained from the Census Bureau. Estimates of the July 1^st^ US population by sex, age, and insurance type were calculated for each study year by applying the average proportion of individuals with private and government health insurance for the 0-17 age group across all age-sex categories. The IRs of IPD and its manifestations in the general US pediatric population were calculated by multiplying the IRs for each age-sex-insurance type group in the MarketScan data with the proportion of that group in the general US pediatric population, and summing across all groups.

## **Interrupted time series estimation**

Generalized linear models (GLM) with negative binomial family and log link function were used to estimate the parameters of the ITS models. The reasons for using GLM models are that they (1) offer flexibility when the outcome variable is constrained, in our case rates, or when errors are non-normally distributed, (2) are a recommended alternative to a log transformation of the outcome variable and (3) retain a similar analysis framework as linear models. The modified Park test was used in choosing the appropriate combination of distributional family and link function. A likelihood ratio test was used to test for overdispersion, and plots of standardized deviance residuals vs. the predicted model counts were examined, suggesting that the negative binomial model provides better fit.

The estimating equations used for each of the data sources are shown in detail below.

Equation used for the models estimated in the commercially insured population (1998-2018):

$$\ln\left( E\left[ N_{i}^{IPD} \right] \right)=\ln\left( N_{i}^{Risk} \right)+\beta_{0}+\beta_{1}T_{i} {+ \beta}_{2}\times EarlyPCV7_{i}+\beta_{3}\times TEarlyPCV7_{i}+\beta_{4}\times LatePCV7_{i}+\beta_{5}\times TLatePCV7_{i}$$

$$+\beta_{6}\times EarlyPCV{13}_{i}+\beta_{7}\times TEarlyPCV{13}_{i}+\beta_{8}\times LatePCV{13}_{i}+ \beta_{9}\times TLatePCV{13}_{i} +\sum_{m=1}^{12} \gamma_{m}{\times I}_{m}$$

where:

$N_{i}^{IPD}$is the episode count in each population stratum for month *i* = 1 to *n;*

$N_{i}^{Risk}$ is the population at risk in each population stratum for each month;

$T_{i}$is a linear time trend indicator, which equals 1 in January 1998 and increases by 1 with each subsequent month;

$EarlyPCV7$ is an indicator which equals 0 before January 2001 and 1 after the start of the early PCV7 period (i.e. from January 2001 onward);

$TEarlyPCV7$ is a linear time trend indicator for the early PCV7 period, which equals 0 before January 2001, then increases linearly each month as 1, 2, 3, … starting in January 2001;

$LatePCV7$ is an indicator which equals 0 before January 2006 and 1 after the start of the late PCV7 period (i.e. from January 2006 onward);

$TLatePCV7$ is a linear time trend indicator for the late PCV7 period, which equals 0 before January 2006 and then increases linearly each month as 1, 2, 3, … starting in January 2006;

$EarlyPCV13$ is an indicator which equals 0 before January 2011 and 1 after the start of the early PCV13 period (i.e. from January 2011 onward);

$TEarlyPCV13$ is a linear time trend indicator for the early PCV13 period, which equals 0 before January 2011 and then increases linearly each month as 1, 2, 3, … starting in January 2011;

$LatePCV13$ is an indicator which equals 0 before January 2014 and 1 after the start of the late PCV13 period (i.e. from January 2014 onward);

$TLatePCV13$ is a linear time trend indicator for the late PCV13 period, which equals 0 before January 2014 and then increases linearly each month as 1, 2, 3, … starting in January 2014;

$I_{m}$ is an indicator for each month *m =* 2 to 12, i.e. February, … December; the indicator for January is excluded and serves as the reference.

Equation used for the Medicaid model (2006-2018):

$$\ln\left( E\left[ N_{i}^{IPD} \right] \right)=\ln\left( N_{i}^{Risk} \right)+\beta_{0}+\beta_{1}T_{i} {+ \beta}_{2}\times EarlyPCV{13}_{i}+\beta_{3}\times TEarlyPCV{13}_{i}+\beta_{4}\times LatePCV{13}_{i}+ \beta_{5}\times TLatePCV{13}_{i}+\sum_{m=1}^{12} \gamma_{m}{\times I}_{m}$$

where:

$N_{i}^{IPD}$is the episode count in each population stratum for month *i* = 1 to *n;*

$N_{i}^{Risk}$ is the population at risk in each population stratum for each month;

$T_{i}$is a linear time trend indicator, which equals 1 in January 2006 and increases by 1 with each subsequent month;

$EarlyPCV13$ is an indicator which equals 0 before January 2011 and 1 after the start of the early PCV13 period (i.e. from January 2011 onward);

$TEarlyPCV13$ is a linear time trend indicator for the early PCV13 period, which equals 0 before January 2011 and then increases each month as 1, 2, 3, … starting in January 2011;

$LatePCV13$ is an indicator which equals 0 before January 2014 and 1 after the start of the late PCV13 period (i.e. from January 2014 onward);

$TLatePCV13$ is a linear time trend indicator for the late PCV13 period, which equals 0 before January 2014 and then increases each month as 1, 2, 3, … starting in January 2014;

$I_{m}$ is an indicator for each month *m =* 2 to 12, i.e. February, … December; the indicator for January is excluded and serves as the reference;

After the ITS models were estimated, autocorrelation of the residuals was examined via autocorrelation function (ACF) and partial autocorrelation function (PACF) plots, confirming that no significant autocorrelation was present. ACF and PACF plots for the ITS models of IPD in commercially insured and Medicaid children are shown in Supplemental Figures A4 and A5, respectively.

## Supplemental Table A2. Size of MarketScan commercially insured children population at risk in person-years and estimates of the the total US pediatric population with commercial insurance (1998-2018)

|  | **Total population at risk in person years (MarketScan) ^1-2^** | | | |  | **Total US commercially insured population estimates^3^** | | | |
| --- | --- | --- | --- | --- | --- | --- | --- | --- | --- |
| **Year** | **All ages** | **Age <2** | **Age 2-4** | **Age 5-17** |  | **All ages** | **Age <2** | **Age 2-4** | **Age 5-17** |
| 1998 | 751,826 | 63,272 | 102,853 | 585,701 |  | 54,848,589 | 6,094,288 | 9,141,431 | 39,612,870 |
| 1999 | 773,498 | 64,818 | 105,906 | 602,773 |  | 55,060,789 | 6,117,865 | 9,176,798 | 39,766,126 |
| 2000 | 822,472 | 68,020 | 114,146 | 640,306 |  | 55,229,651 | 6,136,628 | 9,204,942 | 39,888,082 |
| 2001 | 1,231,275 | 99,473 | 171,619 | 960,182 |  | 53,869,133 | 5,985,459 | 8,978,189 | 38,905,485 |
| 2002 | 2,440,344 | 217,813 | 349,767 | 1,872,764 |  | 53,454,645 | 5,939,405 | 8,909,107 | 38,606,132 |
| 2003 | 3,744,623 | 335,704 | 540,081 | 2,868,838 |  | 51,673,050 | 5,741,450 | 8,612,175 | 37,319,425 |
| 2004 | 4,869,883 | 426,452 | 704,260 | 3,739,172 |  | 51,413,909 | 5,712,657 | 8,568,985 | 37,132,268 |
| 2005 | 5,336,790 | 468,088 | 796,059 | 4,072,643 |  | 51,501,622 | 5,722,402 | 8,583,604 | 37,195,616 |
| 2006 | 6,975,220 | 633,107 | 1,014,292 | 5,327,821 |  | 51,070,885 | 5,674,543 | 8,511,814 | 36,884,528 |
| 2007 | 7,157,315 | 652,795 | 1,044,293 | 5,460,227 |  | 50,573,110 | 5,619,234 | 8,428,852 | 36,525,024 |
| 2008 | 7,501,516 | 671,090 | 1,110,240 | 5,720,185 |  | 49,180,629 | 5,464,514 | 8,196,771 | 35,519,343 |
| 2009 | 8,685,402 | 774,717 | 1,328,672 | 6,582,012 |  | 46,756,619 | 5,195,180 | 7,792,770 | 33,768,669 |
| 2010 | 9,763,080 | 878,663 | 1,438,974 | 7,445,443 |  | 45,445,218 | 5,049,469 | 7,574,203 | 32,821,546 |
| 2011 | 11,022,990 | 986,504 | 1,625,317 | 8,411,169 |  | 44,926,675 | 4,991,853 | 7,487,779 | 32,447,043 |
| 2012 | 10,930,176 | 941,730 | 1,598,470 | 8,389,975 |  | 45,029,374 | 5,003,264 | 7,504,896 | 32,521,215 |
| 2013 | 8,845,721 | 760,576 | 1,309,439 | 6,775,706 |  | 43,621,691 | 4,846,855 | 7,270,282 | 31,504,555 |
| 2014 | 9,343,998 | 837,266 | 1,328,696 | 7,178,036 |  | 42,647,631 | 4,738,626 | 7,107,939 | 30,801,067 |
| 2015 | 5,677,035 | 510,025 | 805,890 | 4,361,120 |  | 43,229,136 | 4,803,237 | 7,204,856 | 31,221,042 |
| 2016 | 5,615,207 | 492,931 | 800,411 | 4,321,865 |  | 43,725,652 | 4,858,406 | 7,287,609 | 31,579,638 |
| 2017 | 5,253,221 | 463,883 | 778,064 | 4,011,273 |  | 44,102,671 | 4,900,297 | 7,350,445 | 31,851,929 |
| 2018 | 5,317,697 | 487,172 | 769,136 | 4,061,389 |  | 42,425,300 | 4,713,922 | 7,070,883 | 30,640,494 |

**Notes:**

[1] Patients' month and day of birth was imputed as July 1st for all patients. Age at onset was calculated as the difference between condition start date and imputed birth date.

[2] An average of 7.08 million commercially-insured children contributed 5.81 million person-years at risk each year.

[3] Data from the US Census Bureau. The number of patients with commercial insurance was estimated using the proportion of patients with employment based insurance vs. Medicaid for the population under 18 years old.

## Supplemental Table A3. Size of MarketScan Medicaid-insured children population at risk in person-years and estimates of the the total US pediatric population with Medicaid coverage (2001-2018)

|  | **Total population at risk in person years ( MarketScan) ^1-2^** | | | |  | **Total US Medicaid-covered population estimates^3^** | | | |
| --- | --- | --- | --- | --- | --- | --- | --- | --- | --- |
| **Year** | **All ages** | **Age <2** | **Age 2-4** | **Age 5-17** |  | **All ages** | **Age <2** | **Age 2-4** | **Age 5-17** |
| 2001 | 1,309,359 | 236,937 | 261,527 | 810,895 |  | 18,758,446 | 2,084,272 | 3,126,408 | 13,547,767 |
| 2002 | 2,095,079 | 360,489 | 409,664 | 1,324,926 |  | 19,857,194 | 2,206,355 | 3,309,532 | 14,341,307 |
| 2003 | 2,453,468 | 400,734 | 483,844 | 1,568,890 |  | 21,907,390 | 2,434,154 | 3,651,232 | 15,822,004 |
| 2004 | 2,581,425 | 400,834 | 512,851 | 1,667,740 |  | 22,377,159 | 2,486,351 | 3,729,526 | 16,161,281 |
| 2005 | 2,813,872 | 436,038 | 566,058 | 1,811,776 |  | 22,483,295 | 2,498,144 | 3,747,216 | 16,237,935 |
| 2006 | 2,526,832 | 413,689 | 483,704 | 1,629,439 |  | 23,030,531 | 2,558,948 | 3,838,422 | 16,633,161 |
| 2007 | 2,406,917 | 408,134 | 456,321 | 1,542,461 |  | 23,829,627 | 2,647,736 | 3,971,604 | 17,210,286 |
| 2008 | 2,574,256 | 430,275 | 501,395 | 1,642,585 |  | 25,329,028 | 2,814,336 | 4,221,505 | 18,293,187 |
| 2009 | 2,983,346 | 479,269 | 614,240 | 1,889,837 |  | 28,282,908 | 3,142,545 | 4,713,818 | 20,426,545 |
| 2010 | 3,139,573 | 486,189 | 634,307 | 2,019,077 |  | 28,850,536 | 3,205,615 | 4,808,423 | 20,836,498 |
| 2011 | 2,898,033 | 426,211 | 581,710 | 1,890,111 |  | 29,181,181 | 3,242,353 | 4,863,530 | 21,075,298 |
| 2012 | 3,947,665 | 537,340 | 779,592 | 2,630,734 |  | 29,157,759 | 3,239,751 | 4,859,626 | 21,058,381 |
| 2013 | 4,032,490 | 531,923 | 787,962 | 2,712,605 |  | 30,331,309 | 3,370,145 | 5,055,218 | 21,905,945 |
| 2014 | 5,343,957 | 712,153 | 971,404 | 3,660,400 |  | 31,271,369 | 3,474,597 | 5,211,895 | 22,584,877 |
| 2015 | 5,722,235 | 732,848 | 1,000,325 | 3,989,062 |  | 30,832,864 | 3,425,874 | 5,138,811 | 22,268,180 |
| 2016 | 5,657,009 | 684,027 | 990,031 | 3,982,951 |  | 30,321,348 | 3,369,039 | 5,053,558 | 21,898,751 |
| 2017 | 5,652,255 | 673,614 | 1,019,899 | 3,958,741 |  | 29,860,329 | 3,317,814 | 4,976,721 | 21,565,793 |
| 2018 | 4,703,874 | 577,989 | 822,895 | 3,302,990 |  | 31,296,069 | 3,477,341 | 5,216,011 | 22,602,716 |

**Notes:**

[1] Patients' month and day of birth was imputed as July 1st for all patients. Age at onset was calculated as the difference between condition start date and imputed birth date.

[2] An average of 4.27 million children contributed 3.49 million person-years at risk each year.

[3] Data from the US Census Bureau. The number of patients with Medicaid was estimated using the proportion of patients with employment based insurance vs. Medicaid for the population under 18 years old.

## Supplemental Table A4. Demographic characteristics of the population at risk (1998-2018)

|  | **Commercially Insured** | | | | | | | | | | **Medicaid** | | | | | | | |
| --- | --- | --- | --- | --- | --- | --- | --- | --- | --- | --- | --- | --- | --- | --- | --- | --- | --- | --- |
|  | **Pre-PCV7** | | **Early PCV7** | | **Late PCV7** | | **Early PCV13** | | **Late PCV13** | | **Early PCV7** | | **Late PCV7** | | **Early PCV13** | | **Late PCV13** | |
|  | **(1998-1999)** | | **(2001-2005)** | | **(2006-2009)** | | **(2011-2013)** | | **(2014-2018)** | | **(2001-2005)** | | **(2006-2009)** | | **(2011-2013)** | | **(2014-2018)** | |
| **Number of PY at risk, N** | **1,525,323** | | **17,622,916** | | **30,319,452** | | **30,798,887** | | **31,207,158** | | **11,253,203** | | **10,491,350** | | **10,878,188** | | **27,079,328** | |
| **Number of children at risk, N** | **1,786,236** | | **21,686,073** | | **37,053,604** | | **37,669,249** | | **37,661,189** | | **14,686,042** | | **13,713,038** | | **13,054,994** | | **31,476,279** | |
| **Age, mean (SD)** | 9.36 | (5.13) | 9.17 | (5.14) | 9.10 | (5.17) | 9.11 | (5.13) | 9.18 | (5.16) | 7.90 | (5.24) | 7.95 | (5.30) | 8.13 | (5.13) | 8.58 | (5.16) |
| 0-1 year, n (%) | 128,090 | (8.4%) | 1,547,530 | (8.8%) | 2,731,709 | (9.0%) | 2,688,810 | (8.7%) | 2,791,277 | (8.9%) | 1,835,031 | (16.3%) | 1,731,368 | (16.5%) | 1,495,474 | (13.7%) | 3,380,631 | (12.5%) |
| 2-4 years, n (%) | 208,759 | (13.7%) | 2,561,786 | (14.5%) | 4,497,497 | (14.8%) | 4,533,226 | (14.7%) | 4,482,198 | (14.4%) | 2,233,944 | (19.9%) | 2,055,660 | (19.6%) | 2,149,265 | (19.8%) | 4,804,554 | (17.7%) |
| 5-17 years, n (%) | 1,188,474 | (77.9%) | 13,513,600 | (76.7%) | 23,090,246 | (76.2%) | 23,576,851 | (76.6%) | 23,933,683 | (76.7%) | 7,184,228 | (63.8%) | 6,704,323 | (63.9%) | 7,233,450 | (66.5%) | 18,894,144 | (69.8%) |
| **Male, n (%)** | 781,982 | (51.3%) | 9,008,536 | (51.1%) | 15,486,019 | (51.1%) | 15,733,677 | (51.1%) | 15,933,139 | (51.1%) | 5,730,324 | (50.9%) | 5,341,057 | (50.9%) | 5,524,379 | (50.8%) | 13,847,263 | (51.1%) |
| **Region** |  |  |  |  |  |  |  |  |  |  |  |  |  |  |  |  |  |  |
| Northeast | 236,547 | (15.5%) | 1,700,260 | (9.6%) | 3,280,655 | (10.8%) | 5,324,554 | (17.3%) | 5,597,806 | (17.9%) | - | - | - | - | - | - | - | - |
| North Central | 361,447 | (23.7%) | 3,855,309 | (21.9%) | 7,944,535 | (26.2%) | 7,389,842 | (24.0%) | 6,640,078 | (21.3%) | - | - | - | - | - | - | - | - |
| South | 671,600 | (44.0%) | 7,408,222 | (42.0%) | 14,189,698 | (46.8%) | 10,620,846 | (34.5%) | 12,769,811 | (40.9%) | - | - | - | - | - | - | - | - |
| West | 91,709 | (6.0%) | 4,415,513 | (25.1%) | 4,699,036 | (15.5%) | 6,651,946 | (21.6%) | 5,834,866 | (18.7%) | - | - | - | - | - | - | - | - |
| Missing/unknown | 164,020 | (10.8%) | 243,612 | (1.4%) | 205,529 | (0.7%) | 811,698 | (2.6%) | 364,597 | (1.2%) | - | - | - | - | - | - | - | - |
| **Urbanicity** |  |  |  |  |  |  |  |  |  |  |  |  |  |  |  |  |  |  |
| Urban, n (%) | 337,203 | (22.1%) | 3,116,716 | (17.7%) | 4,802,730 | (15.8%) | 4,205,925 | (13.7%) | 3,543,028 | (11.4%) | - | - | - | - | - | - | - | - |
| Rural, n (%) | 1,023,769 | (67.1%) | 14,277,841 | (81.0%) | 25,345,433 | (83.6%) | 25,792,188 | (83.7%) | 26,288,879 | (84.2%) | - | - | - | - | - | - | - | - |
| Missing | 164,351 | (10.8%) | 228,359 | (1.3%) | 171,289 | (0.6%) | 800,774 | (2.6%) | 1,375,250 | (4.4%) | - | - | - | - | - | - | - | - |
| **Health plan types** |  |  |  |  |  |  |  |  |  |  |  |  |  |  |  |  |  |  |
| FFS, n (%) | 506,524 | (33.2%) | 1,311,144 | (7.4%) | 624,969 | (2.1%) | 311,885 | (1.0%) | 485,225 | (1.6%) | 4,800,703 | (42.7%) | 3,313,709 | (31.6%) | 3,882,067 | (35.7%) | 9,330,480 | (34.5%) |
| EPO, n (%) | 7,713 | (0.5%) | 132,809 | (0.8%) | 216,797 | (0.7%) | 726,943 | (2.4%) | 336,806 | (1.1%) | 0 | (0.0%) | 0 | (0.0%) | 0 | (0.0%) | 0 | (0.0%) |
| HMO, n (%) | 133,900 | (8.8%) | 4,163,711 | (23.6%) | 4,836,348 | (16.0%) | 3,963,862 | (12.9%) | 3,173,868 | (10.2%) | 3,005,577 | (26.7%) | 6,174,475 | (58.9%) | 6,643,672 | (61.1%) | 17,688,874 | (65.3%) |
| POS, n (%) | 477,683 | (31.3%) | 2,613,797 | (14.8%) | 2,834,579 | (9.3%) | 1,904,164 | (6.2%) | 2,155,477 | (6.9%) | 3,446,166 | (30.6%) | 756,055 | (7.2%) | 343,209 | (3.2%) | 1,038 | (0.0%) |
| PPO, n (%) | 394,230 | (25.8%) | 8,730,598 | (49.5%) | 19,809,845 | (65.3%) | 19,222,249 | (62.4%) | 17,435,433 | (55.9%) | 0 | (0.0%) | 2,794 | (0.0%) | 0 | (0.0%) | 37,901 | (0.1%) |
| CDHP, n (%) | 0 | (0.0%) | 214,158 | (1.2%) | 820,817 | (2.7%) | 1,471,679 | (4.8%) | 3,286,131 | (10.5%) | 0 | (0.0%) | 0 | (0.0%) | 0 | (0.0%) | 0 | (0.0%) |
| HDHP, n (%) | 0 | (0.0%) | 0 | (0.0%) | 126,870 | (0.4%) | 1,446,072 | (4.7%) | 3,219,459 | (10.3%) | 0 | (0.0%) | 0 | (0.0%) | 0 | (0.0%) | 0 | (0.0%) |
| Missing, n (%) | 5,273 | (0.3%) | 456,699 | (2.6%) | 1,049,227 | (3.5%) | 1,752,033 | (5.7%) | 1,114,759 | (3.6%) | 757 | (0.0%) | 244,317 | (2.3%) | 9,239 | (0.1%) | 21,036 | (0.1%) |

**Notes:**

[1] Enrollees' month and day of birth was imputed as July 1st for all patients. Age at onset was calculated as the difference between condition start date and imputed birth date.

[2] Enrollees' demographic characteristics and risk factors were first determined by each calendar year and then combined by PCV periods, assuming each year has distinct enrollee population.

[3] Standard deviations for age in each vaccine period were calculated using the pooled standard deviation of the samples in relevant years.

[4] All values, except for age and number of individuals at risk are reported in person years.

[5] Time periods are defined as follows: Pre PCV7: 1998-1999; Early PCV7: 2001-2005; Late PCV7: 2006-2009; Early PCV13: 2011-2013; Late PCV13: 2014-2018. Years 2000 and 2010 are considered transition years and were excluded from the analysis.

**Abbreviations:** CDHP: Consumer directed health plan; EPO: Exclusive provider organization; FFS: Fee-for-service; HDHP: High-deductible health plan; HMO: Health maintenance organization; PCV: Pneumococcal conjugate vaccine; POS: Point of service; PPO: Preferred provider organization; SD: Standard deviation.

## Supplemental Table A5. Demographic characteristics of Medicaid insured children aged < 18 years with IPD episodes, by PCV period (2001-2018)

|  | **Medicaid** | | | |
| --- | --- | --- | --- | --- |
|  | **Early PCV7** | **Late PCV7** | **Early PCV13** | **Late PCV13** |
|  | **(2001-2005)** | **(2006-2009)** | **(2011-2013)** | **(2014-2018)** |
| **Total number of children with IPD, N** | **1,248** | **1,053** | **632** | **1,058** |
| **Age, mean (SD)** | 2.71 (4.32) | 2.65 (4.35) | 3.66 (4.94) | 4.32 (4.76) |
| <2 years, % | 61.7% | 63.2% | 53.3% | 39.3% |
| 2-4 years, % | 17.2% | 17.3% | 16.3% | 24.0% |
| 5-17 years, % | 21.1% | 19.6% | 30.4% | 36.7% |
| **Male, %** | 54.3% | 53.4% | 54.6% | 58.4% |
| **Health plan types** |  |  |  |  |
| HMO/EPO, % | 15.7% | 59.7% | 57.3% | 63.0% |
| PPO/POS, % | 31.7% | 6.6% | 1.7% | 0.3% |
| FFS, % | 52.5% | 31.1% | 40.8% | 36.4% |
| Missing, % | 0.2% | 2.5% | 0.2% | 0.3% |

**Notes:**

[1] Patients' month and day of birth was imputed as July 1st for all patients. Age at onset was calculated as the difference between condition start date and imputed birth date.

[2] Patients' demographic characteristics and risk factors were firstly determined by each calendar year and then combined by PCV periods, assuming each year has a distinct patient population.

[3] For each calendar year, patients' demographic characteristics were determined at the index episode, which was defined as the first IPD episode in the given calendar year.

[4] Standard deviations for age in each vaccine period were calculated using the pooled standard deviation of the samples in relevant years.

**Abbreviations:** EPO: Exclusive provider organization; FFS: Fee-for-service; HMO: Health maintenance organization; IPD: Invasive pneumococcal disease; PCV: Pneumococcal conjugate vaccine; POS: Point of service; PPO: Preferred provider organization; SD: Standard deviation.

## Supplemental Table A6. Presence of risk factors for pneumococcal disease among IPD patients aged <18 years in the 6 months prior to IPD episodes, 1998-2018

|  | **Commercially Insured** | | | | | | | | | | **Medicaid** | | | | | | | |
| --- | --- | --- | --- | --- | --- | --- | --- | --- | --- | --- | --- | --- | --- | --- | --- | --- | --- | --- |
|  | **Pre-PCV7** | | **Early PCV7** | | **Late PCV7** | | **Early PCV13** | | **Late PCV13** | | **Early PCV7** | | **Late PCV7** | | **Early PCV13** | | **Late PCV13** | |
|  | **(1998-1999)** | | **(2001-2005)** | | **(2006-2009)** | | **(2011-2013)** | | **(2014-2018)** | | **(2001-2005)** | | **(2006-2009)** | | **(2011-2013)** | | **(2014-2018)** | |
| **Total number of IPD patients, N** | **98** | | **438** | | **888** | | **504** | | **560** | | **646** | | **590** | | **412** | | **762** | |
| **Any risk factor, n (%)** | 14 | (14.3%) | 116 | (26.5%) | 223 | (25.1%) | 150 | (29.8%) | 250 | (44.6%) | 201 | (31.1%) | 205 | (34.7%) | 168 | (40.8%) | 427 | (56.0%) |
| Chronic heart disease, n (%) | 1 | (1.0%) | 18 | (4.1%) | 30 | (3.4%) | 24 | (4.8%) | 50 | (8.9%) | 44 | (6.8%) | 42 | (7.1%) | 29 | (7.0%) | 92 | (12.1%) |
| Chronic lung disease including asthma, n (%) | 6 | (6.1%) | 49 | (11.2%) | 116 | (13.1%) | 71 | (14.1%) | 89 | (15.9%) | 96 | (14.9%) | 116 | (19.7%) | 82 | (19.9%) | 170 | (22.3%) |
| Diabetes mellitus, n (%) | 2 | (2.0%) | 3 | (0.7%) | 4 | (0.5%) | 9 | (1.8%) | 11 | (2.0%) | 9 | (1.4%) | 5 | (0.8%) | 5 | (1.2%) | 21 | (2.8%) |
| Cerebrospinal fluid leaks, n (%) | 0 | (0.0%) | 0 | (0.0%) | 0 | (0.0%) | 3 | (0.6%) | 5 | (0.9%) | 1 | (0.2%) | 4 | (0.7%) | 2 | (0.5%) | 6 | (0.8%) |
| Cochlear implant(s), n (%) | 0 | (0.0%) | 1 | (0.2%) | 2 | (0.2%) | 2 | (0.4%) | 0 | (0.0%) | 0 | (0.0%) | 0 | (0.0%) | 0 | (0.0%) | 1 | (0.1%) |
| Sickle cell disease or other hemoglobinopathies,  and anatomic or functional asplenia, n (%) | 0 | (0.0%) | 6 | (1.4%) | 24 | (2.7%) | 21 | (4.2%) | 29 | (5.2%) | 53 | (8.2%) | 49 | (8.3%) | 39 | (9.5%) | 73 | (9.6%) |
| Congenital or acquired immunodeficiency, n (%) | 1 | (1.0%) | 23 | (5.3%) | 32 | (3.6%) | 26 | (5.2%) | 97 | (17.3%) | 36 | (5.6%) | 41 | (6.9%) | 38 | (9.2%) | 149 | (19.6%) |
| HIV infection, n (%) | 0 | (0.0%) | 1 | (0.2%) | 0 | (0.0%) | 0 | (0.0%) | 0 | (0.0%) | 9 | (1.4%) | 2 | (0.3%) | 0 | (0.0%) | 0 | (0.0%) |
| Chronic renal failure or nephrotic syndrome, n (%) | 3 | (3.1%) | 19 | (4.3%) | 15 | (1.7%) | 18 | (3.6%) | 11 | (2.0%) | 18 | (2.8%) | 9 | (1.5%) | 15 | (3.6%) | 15 | (2.0%) |
| Cancer and iatrogenic immunosuppression,  including radiation therapy, n (%) | 5 | (5.1%) | 40 | (9.1%) | 60 | (6.8%) | 45 | (8.9%) | 82 | (14.6%) | 52 | (8.0%) | 49 | (8.3%) | 63 | (15.3%) | 162 | (21.3%) |
| Solid organ transplant, n (%) | 0 | (0.0%) | 10 | (2.3%) | 21 | (2.4%) | 13 | (2.6%) | 43 | (7.7%) | 31 | (4.8%) | 20 | (3.4%) | 18 | (4.4%) | 56 | (7.3%) |
| Alcoholism, n (%) | 0 | (0.0%) | 0 | (0.0%) | 0 | (0.0%) | 1 | (0.2%) | 0 | (0.0%) | 0 | (0.0%) | 0 | (0.0%) | 0 | (0.0%) | 1 | (0.1%) |
| Chronic liver disease, n (%) | 0 | (0.0%) | 6 | (1.4%) | 10 | (1.1%) | 5 | (1.0%) | 18 | (3.2%) | 16 | (2.5%) | 11 | (1.9%) | 11 | (2.7%) | 38 | (5.0%) |
| Multiple myeloma, n (%) | 0 | (0.0%) | 1 | (0.2%) | 0 | (0.0%) | 0 | (0.0%) | 0 | (0.0%) | 0 | (0.0%) | 0 | (0.0%) | 0 | (0.0%) | 0 | (0.0%) |
| Tobacco use, n (%) | 0 | (0.0%) | 1 | (0.2%) | 0 | (0.0%) | 2 | (0.4%) | 0 | (0.0%) | 0 | (0.0%) | 1 | (0.2%) | 1 | (0.2%) | 2 | (0.3%) |
| Hearing loss, n (%) | 0 | (0.0%) | 1 | (0.2%) | 6 | (0.7%) | 4 | (0.8%) | 6 | (1.1%) | 1 | (0.2%) | 2 | (0.3%) | 2 | (0.5%) | 14 | (1.8%) |
| Pre-term birth, n (%) | 0 | (0.0%) | 4 | (0.9%) | 9 | (1.0%) | 3 | (0.6%) | 9 | (1.6%) | 15 | (2.3%) | 10 | (1.7%) | 4 | (1.0%) | 11 | (1.4%) |

**Notes:**

[1] Patients' month and day of birth was imputed as July 1st for all patients. Age at onset was calculated as the difference between condition start date and imputed birth date.

[2] Patients' risk factors were firstly determined by each calendar year and then combined by PCV periods, assuming each year has distinct patient population.

[3] For each calendar year, the index episode was defined as the first IPD episode in the given calendar year.

[4] Patients were required to be continuously enrolled in the health plan for at least 6-months prior to the start of index episode.

[5] For each calendar year, risk factors were determined based on medical claims during the 6-months pre-index period.

**Abbreviations:** HIV: Human Immunodeficiency Virus; IPD: Invasive pneumococcal disease; PCV: Pneumococcal conjugate vaccine.

## Supplemental Table A7. Incidence rates and 95% confidence intervals of unspecified meningitis, bacteremia, bacteremic pneumonia, and other IPD, among Medicaid insured children, in episodes per 100,000 PY (2001-2018)

|  | **All ages** | | | | | **Ages <2** | | | | |
| --- | --- | --- | --- | --- | --- | --- | --- | --- | --- | --- |
| **Period** | **Overall IPD** | **Meningitis** | **Bacteremia** | **Bacteremic pneumonia** | **Other IPD** | **Overall IPD** | **Meningitis** | **Bacteremia** | **Bacteremic pneumonia** | **Other IPD** |
| Early PCV7 | 11.34 (10.73; 11.98) | 1.56 (1.34; 1.80) | 6.50 (6.04; 6.98) | 2.84 (2.55; 3.17) | 0.44 (0.34; 0.59) | 42.62 (39.73; 45.71) | 5.67 (4.68; 6.87) | 28.06 (25.74; 30.60) | 8.39 (7.17; 9.83) | 0.49 (0.26; 0.93) |
| Late PCV7 | 10.33 (9.74; 10.97) | 1.27 (1.07; 1.50) | 5.54 (5.11; 6.01) | 3.15 (2.83; 3.51) | 0.37 (0.27; 0.51) | 39.39 (36.54; 42.46) | 4.27 (3.40; 5.37) | 25.01 (22.76; 27.48) | 9.36 (8.02; 10.91) | 0.75 (0.44; 1.28) |
| Early PCV13 | 6.09 (5.64; 6.57) | 0.93 (0.76; 1.13) | 3.19 (2.87; 3.54) | 1.77 (1.54; 2.04) | 0.19 (0.13; 0.30) | 23.54 (21.20; 26.13) | 3.01 (2.25; 4.03) | 14.98 (13.14; 17.07) | 4.95 (3.94; 6.21) | 0.60 (0.32; 1.14) |
| Late PCV13 | 4.17 (3.94; 4.42) | 0.89 (0.79; 1.01) | 1.88 (1.72; 2.05) | 1.23 (1.11; 1.37) | 0.17 (0.13; 0.23) | 12.81 (11.66; 14.07) | 2.13 (1.69; 2.68) | 7.01 (6.17; 7.96) | 3.05 (2.51; 3.69) | 0.62 (0.41; 0.95) |
|  | **Ages 2-4** | | | | | **Ages 5-17** | | | | |
|  | **Overall IPD** | **Meningitis** | **Bacteremia** | **Bacteremic pneumonia** | **Other IPD** | **Overall IPD** | **Meningitis** | **Bacteremia** | **Bacteremic pneumonia** | **Other IPD** |
| Early PCV7 | 9.76 (8.55; 11.14) | 0.76 (0.48; 1.22) | 5.55 (4.66; 6.62) | 3.27 (2.60; 4.11) | 0.18 (0.07; 0.46) | 3.84 (3.41; 4.32) | 0.75 (0.58; 0.98) | 1.28 (1.04; 1.57) | 1.29 (1.06; 1.59) | 0.52 (0.37; 0.71) |
| Late PCV7 | 9.10 (7.88; 10.50) | 0.97 (0.63; 1.50) | 3.75 (3.00; 4.68) | 4.23 (3.43; 5.22) | 0.15 (0.05; 0.43) | 3.21 (2.81; 3.67) | 0.58 (0.43; 0.80) | 1.06 (0.84; 1.34) | 1.22 (0.99; 1.52) | 0.34 (0.23; 0.51) |
| Early PCV13 | 5.02 (4.16; 6.07) | 0.79 (0.49; 1.27) | 2.51 (1.93; 3.28) | 1.63 (1.17; 2.26) | 0.09 (0.03; 0.34) | 2.79 (2.43; 3.21) | 0.54 (0.39; 0.74) | 0.95 (0.75; 1.21) | 1.16 (0.94; 1.44) | 0.14 (0.08; 0.25) |
| Late PCV13 | 5.62 (4.99; 6.33) | 1.14 (0.88; 1.49) | 2.33 (1.94; 2.80) | 2.10 (1.73; 2.55) | 0.04 (0.01; 0.15) | 2.26 (2.06; 2.48) | 0.61 (0.51; 0.73) | 0.84 (0.72; 0.98) | 0.69 (0.58; 0.82) | 0.12 (0.08; 0.18) |

**Notes:**

[1] Confidence intervals were calculated using the Pearson method.
[2] Time periods are defined as follows: Early PCV7: 2001-2005; Late PCV7: 2006-2009; Early PCV13: 2011-2013; Late PCV13: 2014-2018. Year 2010 is considered transition years and was excluded.

**Abbreviations:** IPD: Invasive pneumococcal disease; PY: Person-years.

## Supplemental Table A8. Estimates from ITS analysis of monthly IPD episode IRs for the Medicaid-insured children aged <18 years (2006-2018)

|  |  | **All ages** | | **Ages <2** | | **Ages 2-4** | | **Ages 5-17** | |  |
| --- | --- | --- | --- | --- | --- | --- | --- | --- | --- | --- |
| **Period** | **IRR** | **IRR**  **(95% CI)** | **p-value** | **IRR**  **(95% CI)** | **p-value** | **IRR**  **(95% CI)** | **p-value** | **IRR**  **(95% CI)** | **p-value** | |
| Late PCV7 | Base Trend | 1.010  (1.004 - 1.016) | 0.002** | 1.015  (1.006 - 1.023) | 0.001** | 1.008  (1.001 - 1.015) | 0.024* | 1.000  (0.990 - 1.009) | 0.928 | |
| Early PCV13 | Change in Level | 0.461  (0.329 - 0.647) | 0.001*** | 0.296  (0.197 - 0.446) | 0.001*** | 0.577  (0.283 - 1.178) | 0.131 | 1.134  (0.695 - 1.850) | 0.616 | |
|  | Change in Trend | 0.991  (0.979 - 1.003) | 0.151 | 0.998  (0.983 - 1.012) | 0.745 | 0.985  (0.963 - 1.009) | 0.220 | 0.992  (0.978 - 1.008) | 0.326 | |
| Late PCV13 | Change in Level | 0.783  (0.592 - 1.036) | 0.087 | 0.619  (0.432 - 0.888) | 0.009** | 1.389  (0.861 - 2.241) | 0.178 | 0.830  (0.572 - 1.204) | 0.326 | |
|  | Change in Trend | 0.994  (0.983 - 1.006) | 0.334 | 0.976  (0.963 - 0.990) | 0.001** | 1.003  (0.981 - 1.026) | 0.786 | 1.012  (0.997 - 1.026) | 0.117 | |

**Abbreviations:** CI: confidence internal; IPD: invasive pneumococcal disease; IRR: incidence rate ratio; ITS: interrupted time series; PCV: pneumococcal conjugate vaccine.

**Notes:**

^a^ Significance codes: ∗ p<0.05; ∗∗ p<0.01; ∗∗∗p< 0.001. P-values less than 0.001 are shown as 0.001***.

^b^ All coefficients were obtained through a negative binomial model with a log link, controlling for seasonality using monthly indicators. IRRs represent the exponentiated regression coefficients and indicate a multiplicative change. Model intercepts are not shown.

^c^ Time periods are defined as follows: Late PCV7: 2006-2009; Early PCV13: 2011-2013; Late PCV13: 2014-2018. Years 2000 and 2010 are considered transition years and were excluded from the model.

^d^ Confidence intervals have been adjusted for heteroscedasticity.

## Supplemental Figure A1. Annual incidence rates of IPD episodes in Medicaid insured children by age group, in episodes per 100,000 PY (2001-2018)


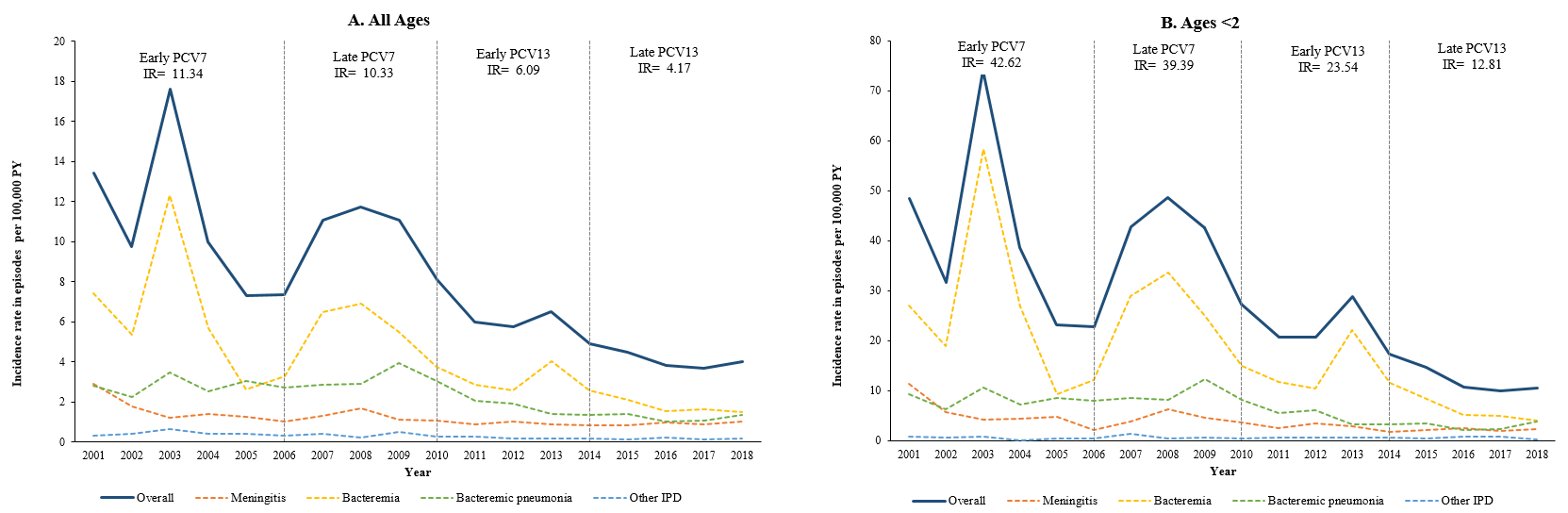


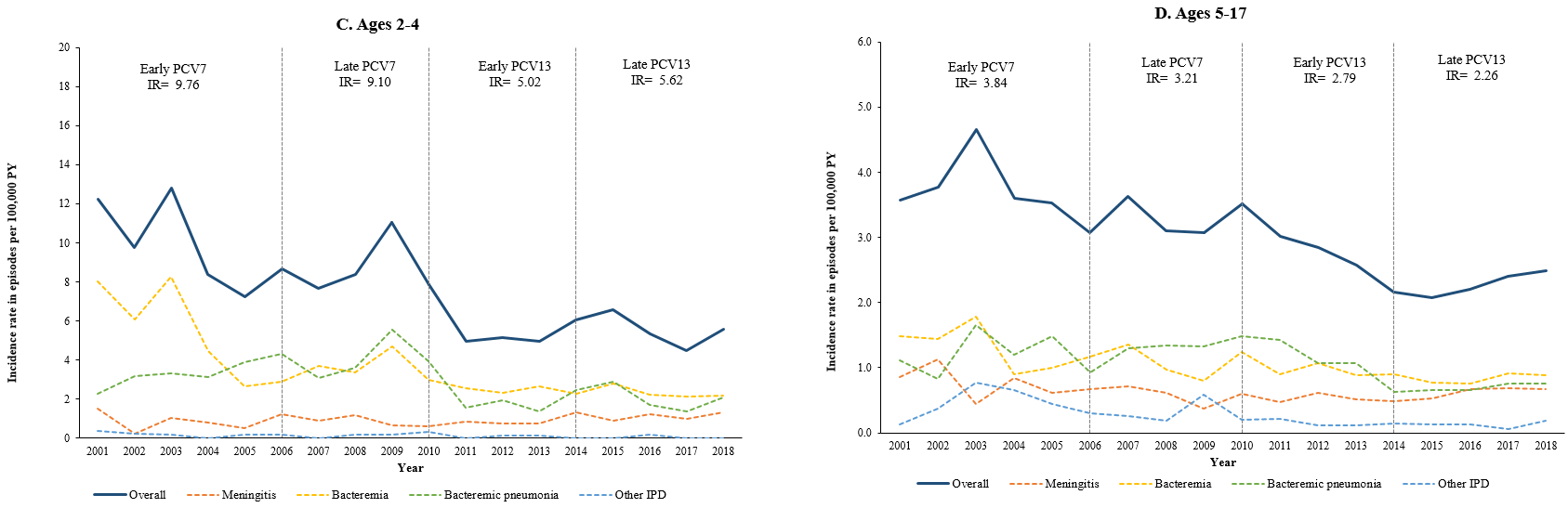


**Note:**

[1] Simple and recurrent episodes were categorized in patients with at least 12 months of continuous health plan enrollment prior to the index episode.

**Abbreviations:** IPD: Invasive pneumococcal disease; IR: Incidence rate; PCV: Pneumococcal conjugate vaccine; PY: Person-years.

## Supplemental Figure A2. Monthly incidence rates and linear time trends predicted from the ITS models in the commercially insured population aged <18 years, in episodes per 100,000 PY (1998-2018)

**A. All ages**


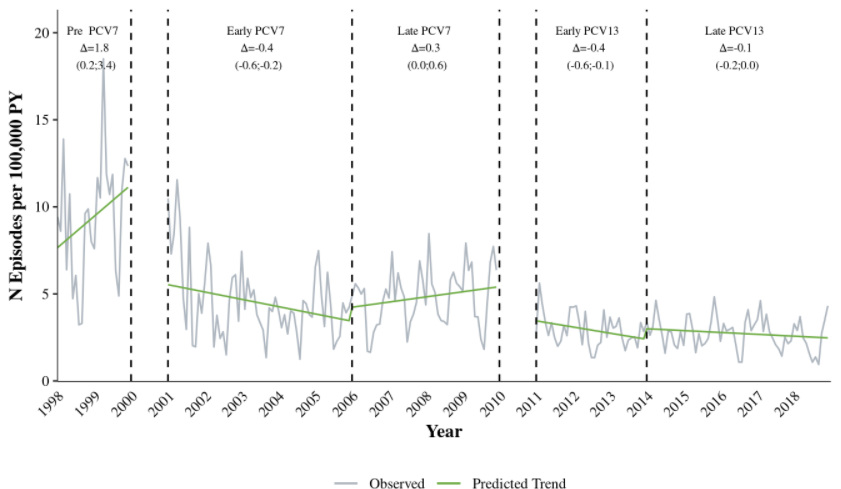

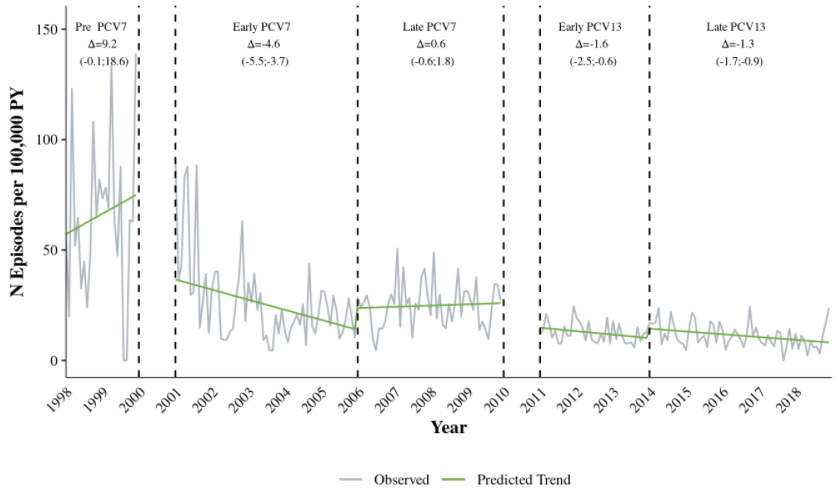

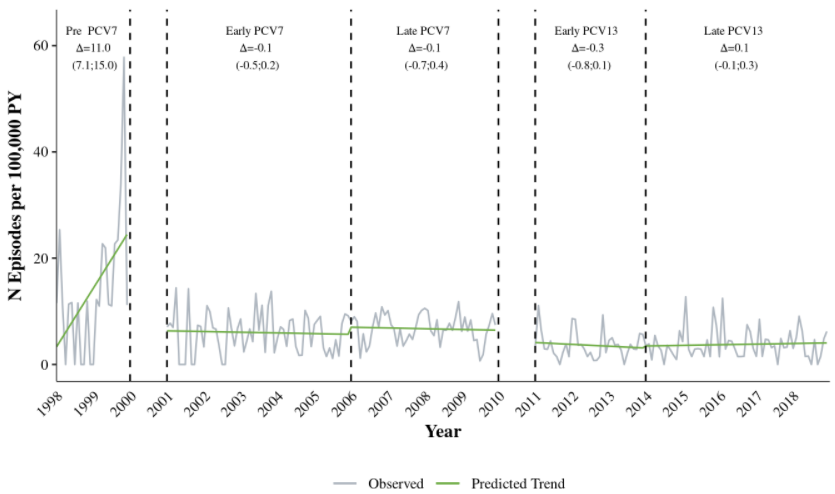

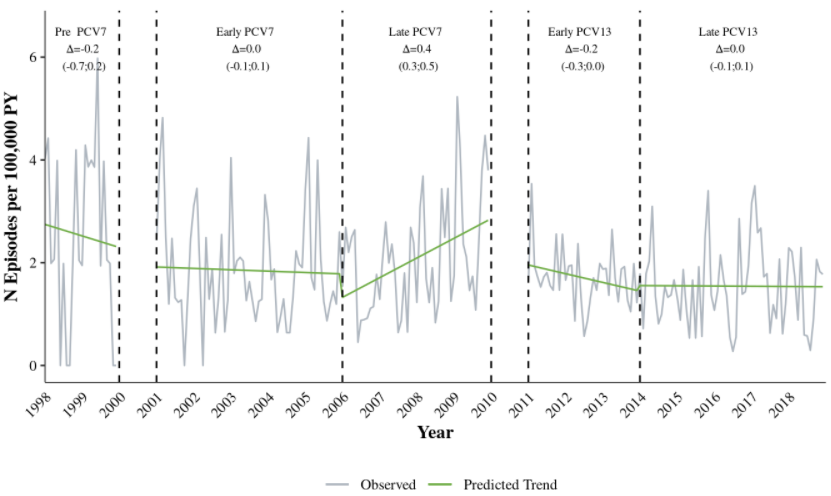


**B. Ages <2**

**C. Ages 2-4**

**D. Ages 5-17**

**Notes:**

[1] Predicted values for episode counts were obtained using a negative binomial GLM model with a log link, controlling for seasonality using monthly indicators. These values were then used to calculate linear trends in incidence rates (green lines). Observed monthly incidence rates are shown in gray.

[2] Time periods are defined as follows: Pre-PCV7: 1998-1999; Early PCV7: 2001-2005; Late PCV7: 2006-2009; Early PCV13: 2011-2013; Late PCV13: 2014-2018. Years 2000 and 2010 are considered transition years and were excluded from the model.

**Abbreviations:** GLM: Generalized linear model; IPD: Invasive pneumococcal disease; PCV: Pneumococcal conjugate vaccine; PY: Person-years.

**Notes:**

[1] Predicted values for IPD episode counts were obtained using a negative binomial GLM model with a log link, controlling for seasonality using monthly indicators. These values were then used to calculate linear trends in incidence rates (green lines). Observed monthly incidence rates are shown in gray.

[2] Time periods are defined as follows: Pre-PCV7: 1998-1999; Early PCV7: 2001-2005; Late PCV7: 2006-2009; Early PCV13: 2011-2013; Late PCV13: 2014-2018. Years 2000 and 2010 are considered transition years and were excluded from the model.

**Abbreviations:** GLM: Generalized linear model; IPD: Invasive pneumococcal disease; PCV: Pneumococcal conjugate vaccine; PY: Person-years.

## Supplemental Figure A3. Monthly incidence rates and linear time trends predicted from the ITS models in the Medicaid-insured population aged <18 years, in episodes per 100,000 PY (2006-2018)


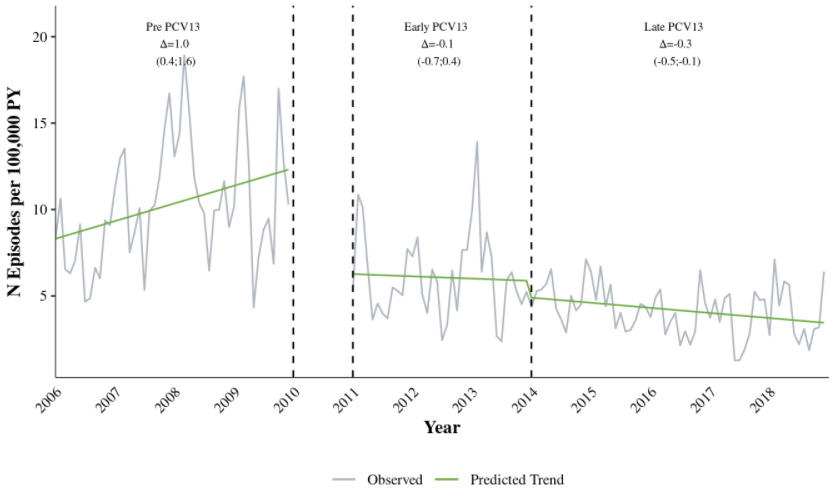

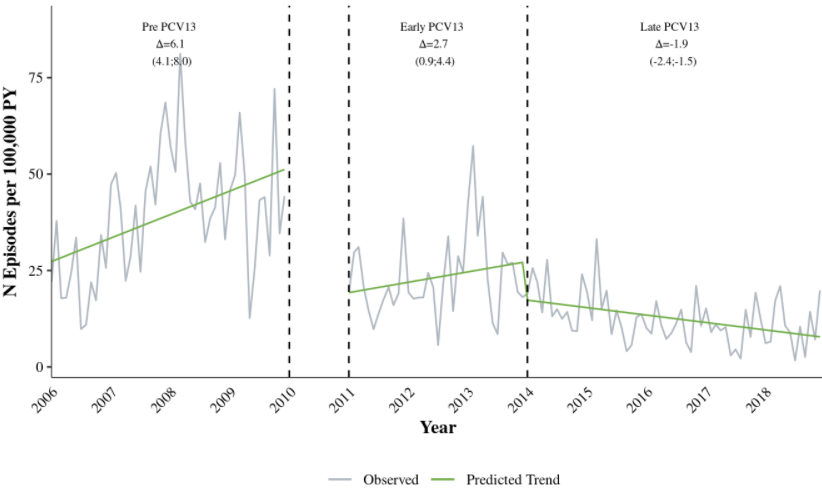

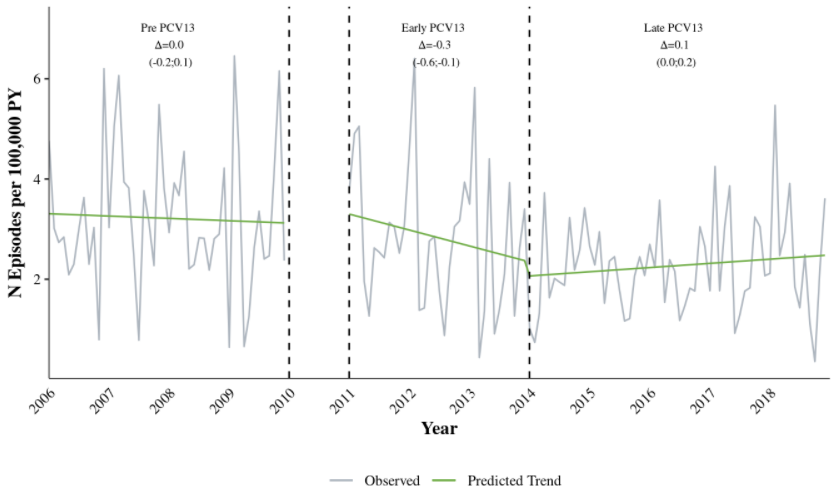

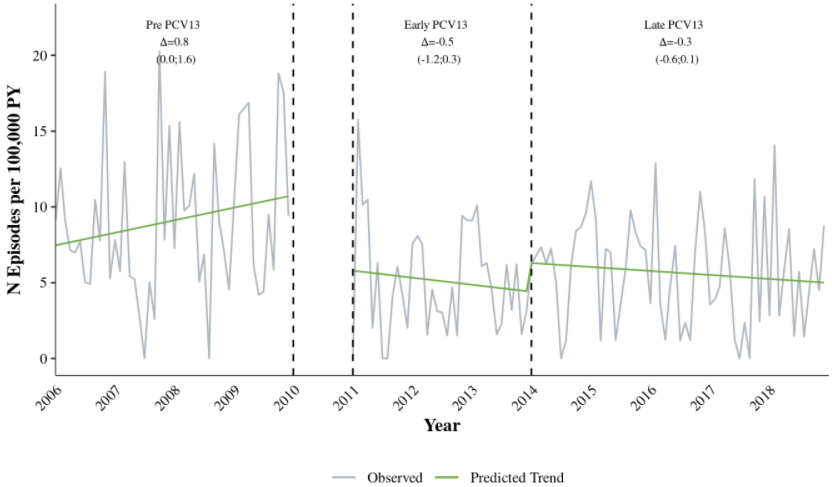


**A. All ages**

**B. Ages <2**

**C. Ages 2-4**

**D. Ages 5-17**

**Notes:**

[1] Predicted values for IPD episode counts were obtained using a negative binomial GLM model with a log link , controlling for seasonality using monthly indicators. These values were then used to calculate linear trends in incidence rates (green lines). Observed monthly incidence rates are shown in gray.

[2] Time periods are defined as follows: Late PCV7: 2006-2009; Early PCV13: 2011-2013; Late PCV13: 2014-2018. Year 2010 is considered transition year and was excluded from the model.

**Abbreviations:** GLM: Generalized linear model; IPD: Invasive pneumococcal disease; PCV: Pneumococcal conjugate vaccine; PY: Person-years.

Supplemental Figure A4. ACF and PACF of the residuals from the ITS model of IPD among commercially insured children aged 0-17 years (1998-2018)


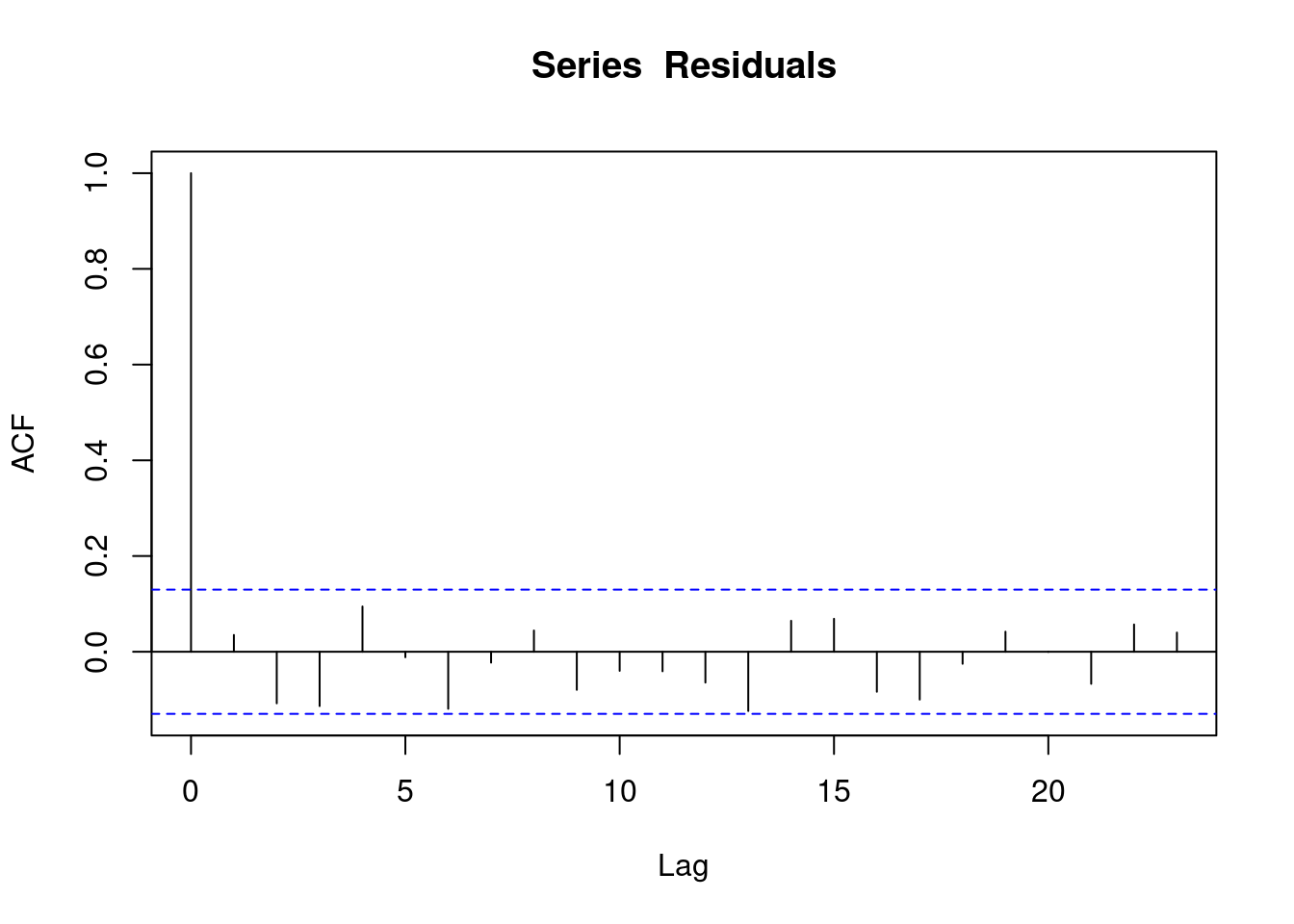

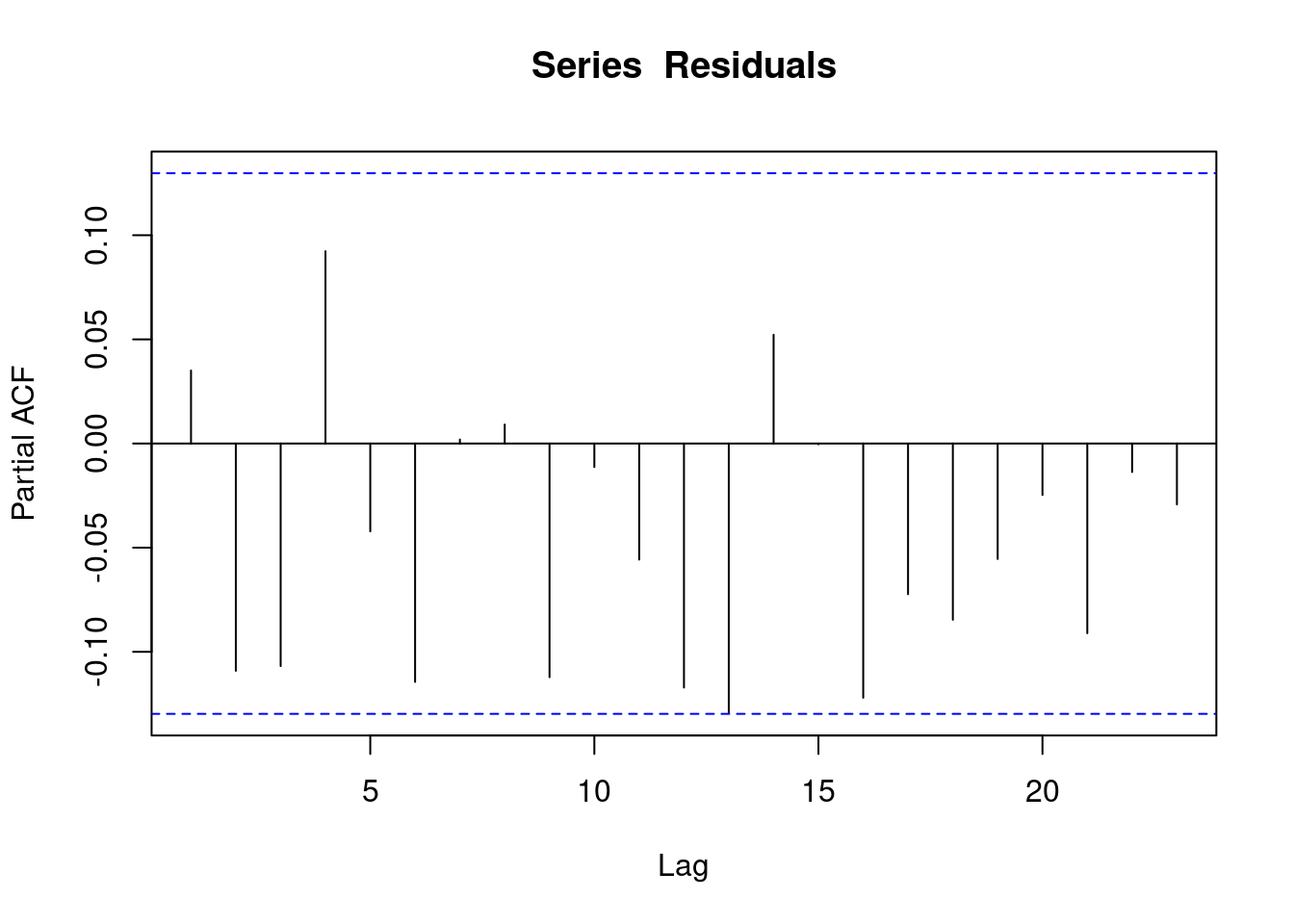


Supplemental Figure A5. ACF and PACF of the residuals from the ITS model of IPD among Medicaid insured children aged 0-17 years (2006-2018).


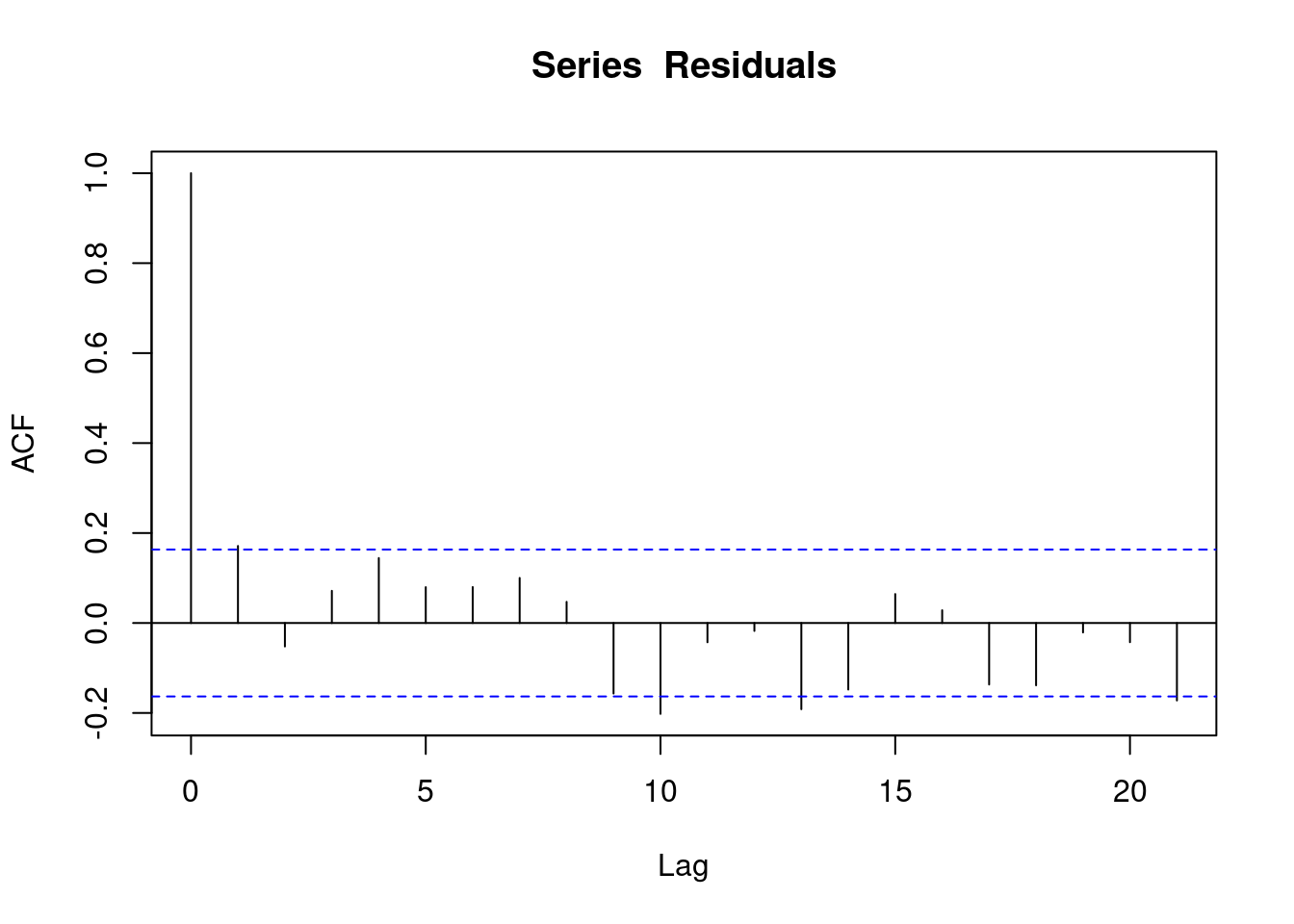

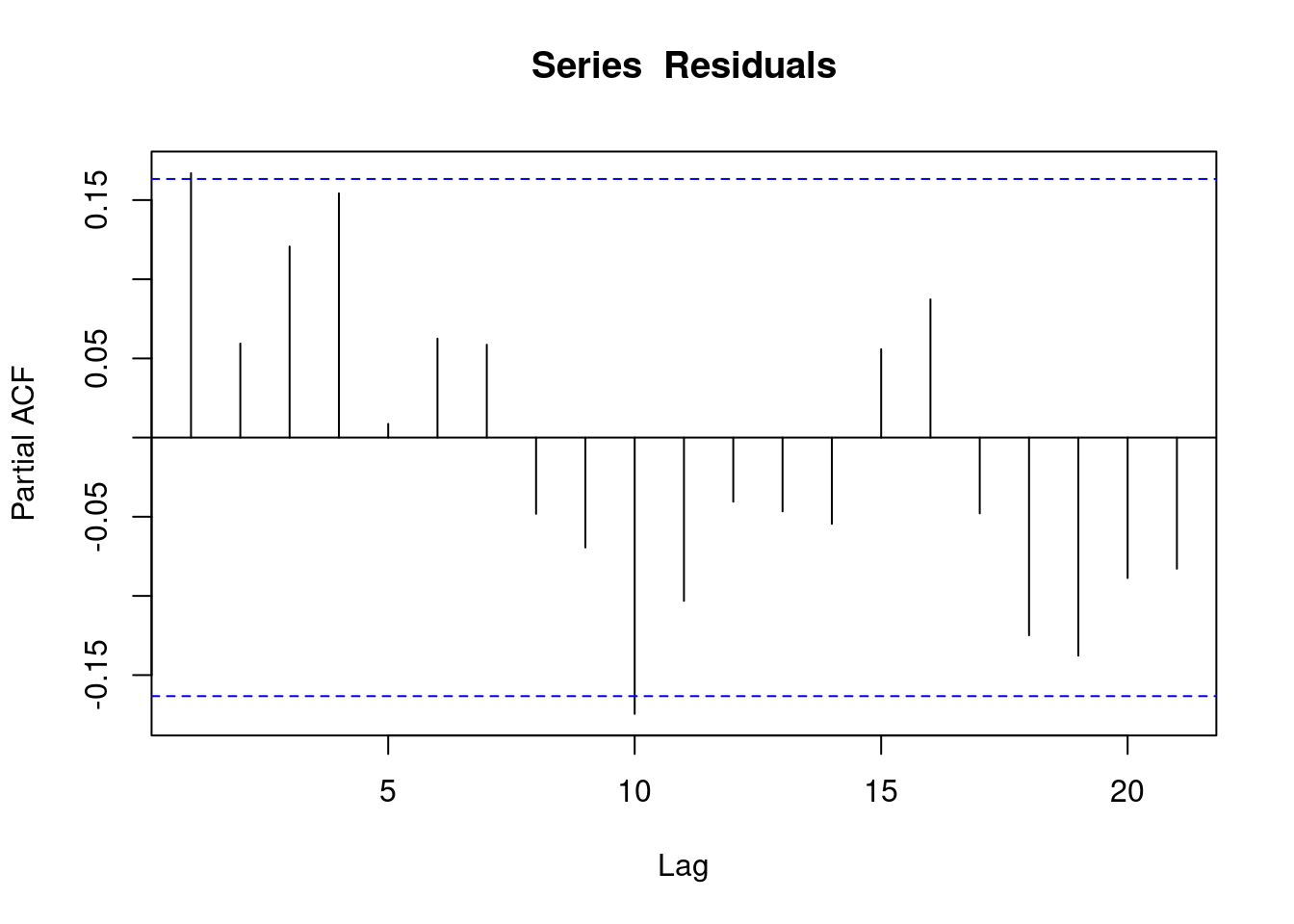

Supplement: Supplementary file 1 — Additional file 1: Supplemental Table A1. Diagnosis and procedure codes used in the study. Supplemental Table A2. Size of MarketScan commercially insured children population at risk in person-years and estimates of the the total US pediatric population with commercial insurance (1998-2018). Supplemental Table A3. Size of MarketScan Medicaid-insured children population at risk in person-years and estimates of the the total US pediatric population with Medicaid coverage (2001-2018). Supplemental Table A4. Demographic characteristics of the population at risk (1998-2018). Supplemental Table A5. Demographic characteristics of Medicaid insured children aged < 18 years with IPD episodes, by PCV period (2001-2018). Supplemental Table A6. Presence of risk factors for pneumococcal disease among IPD patients aged <18 years in the 6 months prior to IPD episodes, 1998-2018. Supplemental Table A7. Incidence rates and 95% confidence intervals of unspecified meningitis, bacteremia, bacteremic pneumonia, and other IPD, among Medicaid insured children, in episodes per 100,000 PY (2001-2018). Supplemental Table A8. Estimates from ITS analysis of monthly IPD episode IRs for the Medicaid-insured children aged <18 years (2006-2018). Supplemental Figure A1. Annual incidence rates of IPD episodes in Medicaid insured children by age group, in episodes per 100,000 PY (2001-2018). Supplemental Figure A2. Monthly incidence rates and linear time trends predicted from the ITS models in the commercially insured population aged <18 years, in episodes per 100,000 PY (1998-2018). Supplemental Figure A3. Monthly incidence rates and linear time trends predicted from the ITS models in the Medicaid-insured population aged <18 years, in episodes per 100,000 PY (2006-2018). Supplemental Figure A4. ACF and PACF of the residuals from the ITS model of IPD among commercially insured children aged 0-17 years (1998-2018). Supplemental Figure A5. ACF and PACF of the residuals from the ITS model of IPD among Med [file 12889_2022_14051_MOESM1_ESM.docx]
